# Theoretical evidence that root penetration ability interacts with soil compaction regimes to affect nitrate capture

**DOI:** 10.1093/aob/mcab144

**Published:** 2021-11-30

**Authors:** Christopher F Strock, Harini Rangarajan, Christopher K Black, Ernst D Schäfer, Jonathan P Lynch

**Affiliations:** Department of Plant Science, The Pennsylvania State University, University Park, PA 16802, USA

**Keywords:** Axial roots, bulk density, lateral roots, leaching, nitrate, OpenSimRoot, root elongation, root system architectural models, soil compaction, soil impedance, soil strength, *Zea mays*

## Abstract

**Background and Aims:**

Although root penetration of strong soils has been intensively studied at the scale of individual root axes, interactions between soil physical properties and soil foraging by whole plants are less clear. Here we investigate how variation in the penetration ability of distinct root classes and bulk density profiles common to real-world soils interact to affect soil foraging strategies.

**Methods:**

We utilize the functional–structural plant model ‘OpenSimRoot’ to simulate the growth of maize (*Zea mays*) root systems with variable penetration ability of axial and lateral roots in soils with (1) uniform bulk density, (2) plow pans and (3) increasing bulk density with depth. We also modify the availability and leaching of nitrate to uncover reciprocal interactions between these factors and the capture of mobile resources.

**Key Results:**

Soils with plow pans and bulk density gradients affected overall size, distribution and carbon costs of the root system. Soils with high bulk density at depth impeded rooting depth and reduced leaching of nitrate, thereby improving the coincidence of nitrogen and root length. While increasing penetration ability of either axial or lateral root classes produced root systems of comparable net length, improved penetration of axial roots increased allocation of root length in deeper soil, thereby amplifying N acquisition and shoot biomass. Although enhanced penetration ability of both root classes was associated with greater root system carbon costs, the benefit to plant fitness from improved soil exploration and resource capture offset these.

**Conclusions:**

While lateral roots comprise the bulk of root length, axial roots function as a scaffold determining the distribution of these laterals. In soils with high soil strength and leaching, root systems with enhanced penetration ability of axial roots have greater distribution of root length at depth, thereby improving capture of mobile resources.

## INTRODUCTION

Roots forage for resources in a dynamic and heterogeneously structured matrix where penetration resistance, or soil strength, is a principal attribute determining both the accessibility and the energetic cost of soil resource acquisition. Mechanical impedance to root growth occurs where there is reduced pore connectivity and space to accommodate the displacement of soil particles by the root apex. This impedance of root elongation is largely dependent upon soil texture, soil water content and bulk density (Hamza and Anderson, 2005; Batey, 2009; Valentine *et al.*, 2012; Suzuki *et al.*, 2013; Hernandez-Ramirez *et al.*, 2014). Suppression of root emergence and elongation due to mechanical impedance restricts soil exploration, soil resource capture and, therefore, the productivity of agroecosystems (Yamaguchi and Tanaka, 1990; Merotto and Mundstock, 1999; Lipiec and Hatano, 2003; Batey, 2009). Beyond directly impeding root growth, conditions that increase soil strength also affect soil hydraulic conductivity, soil water storage capacity, gas diffusivity and permeability, nutrient cycling and the habitat of soil organisms (Tracy *et al.*, 2011; Keller *et al.*, 2019).

Generally, in both monocot and dicot species, mechanical impedance >2 MPa is sufficient to inhibit root elongation, as well as the emergence of new axial and lateral roots (Atwell, 1993; Coelho *et al.*, 2013; Grzesiak *et al*., 2013, 2014; Pfeifer *et al.*, 2014; Colombi and Walter, 2016). In response to mechanical impedance, an increase in root diameter (Logsdon *et al.*, 1987; Atwell, 1993; Pritchard, 1994; Iijima *et al.*, 2000; Tracy *et al*., 2012, 2013; Pfeifer *et al.*, 2014; Colombi and Walter, 2016; Colombi and Keller, 2019; Vanhees *et al.*, 2020), a decrease in the rate of cell division (Clark *et al.*, 2003), reduction in the flux of cells out of the meristem (Croser *et al.*, 1999) and subsequent deceleration of root elongation rates (Atwell, 1993; Gregory, 2006) have been reported across a range of species. Increased root diameter under mechanical impedance is widely framed as an adaptive response as it increases resistance to buckling (Whiteley *et al.*, 1982; Clark *et al.*, 2003; Chimungu *et al.*, 2015), reduces stress at the root apex by deforming the soil near the root tip (Abdalla *et al.*, 1969; Hettiaratchi, 1990; Atwell, 1993; Pritchard, 1994; Kirby and Bengough, 2002; Bengough *et al.*, 2006; Gregory, 2006), and helps displace larger soil particles and aggregates (Whiteley and Dexter, 1984). However, it has recently been reported in maize that genotypes with roots that had less radial expansion in response to mechanical impedance were more likely to cross a compacted layer than those that did increase their diameter (Vanhees *et al*., 2020, 2021). Additionally, other work suggests that root anatomy traits such as cortical cell wall thickness, cortical cell count, cortical cell wall area and stele diameter are better predictors of root penetration ability than root diameter (Chimungu *et al.*, 2015; Vanhees *et al.*, 2020; Schneider *et al.*, 2021*a*). Nevertheless, although increasing root thickness and the expenditure of mucilage and root cap cells may facilitate penetration of individual root axes, these alterations come at an increase in the construction costs of producing and maintaining root length, ultimately affecting the metabolic expenditure of soil exploration (Atwell, 1990; Iijima *et al.*, 2003; Bengough *et al.*, 2011; Ruiz *et al*., 2015, 2016; Colombi and Keller, 2019).

Most studies focused on root penetration of strong soils only consider seedling roots and, consequently, knowledge of how penetration ability varies among mature root classes is limited. Characterization of the penetration ability of different root classes is relevant to understanding the broader soil foraging strategies of the root system. Distinct root classes are, in part, defined by differences in diameter, resulting in variable penetration abilities and anatomy that also affect capacity for nutrient and water uptake (Ahmed *et al.*, 2018; Schneider *et al.*, 2020, Lynch *et al.*, 2021). Where investigated, variation in penetration ability of different root classes has been reported. For example, the effect of root anatomy on penetration ability was observed to be proportionally greater in thinner root classes of maize compared with thicker root classes (Vanhees *et al.*, 2020). Another study with wheat revealed that root anatomy traits were affected by soil strength in a root class-dependent manner (Colombi *et al.*, 2017). Controlling for root diameter, a study by Misra (1997) found that primary roots are capable of 6–32 % greater maximum axial growth force compared with lateral roots of pea. Consequently, class-specific variation in penetration ability may have significant effects on the spatio-temporal distribution of root foraging and acquisition of soil resources. For example, while penetration ability of lateral roots may be important as this root class constitutes the bulk of root length in most angiosperms (Costa *et al.*, 2002; Zobel *et al.*, 2007; Schneider *et al.*, 2017), the penetration ability of axial roots may ultimately determine the spatial and temporal distribution of these higher order lateral roots with depth.

Although many of the processes surrounding the penetration of individual root axes are well researched, the broader interactions between soil structure, metabolic costs of soil exploration and plant fitness are less clear. Presently, empirical data relating soil structure and root length density are sparse due to the complex and laborious nature of the field-based measurements required to adequately assess these interactions. Real-world circumstances where soil structure may modify root length distribution include drought-prone environments with drying topsoil (de Moraes *et al.*, 2018) and no-till systems with high bulk density topsoil (Ehlers *et al.*, 1983), both of which have greater mechanical impedance to root growth in shallow horizons. Additionally, in most soils, the mass of surface soil layers increases bulk density and penetration resistance with depth (Gao *et al.*, 2016). In agricultural soils subject to conventional tillage, mechanical impedance to root growth also commonly occurs in the form of a plow pan just below the depth of tillage. While the ability of individual root axes to penetrate strong soils is related to improved performance under drought (Acuna *et al.*, 2012), scaling our understanding of root penetration ability to the broader interactions between root architecture and soil structure is needed if we are to apply this knowledge to improving the productivity of agroecosystems.

To address questions surrounding the interface between soil structure and soil foraging strategies, functional–structural plant models that combine 3-D representations of plant architecture with physiological models can provide useful insights into such systems, which are challenging to study empirically (Vos *et al.*, 2010; Dunbabin *et al.*, 2013). Plant growth models have historically used very simple representations of soil structure because of uncertainties about how to represent the complexity of the soil matrix, but recent developments in soil modelling have relaxed this limitation and are now ready to be adopted by plant growth models (Postma and Black, 2021). ‘OpenSimRoot’ is an open-source, functional–structural model of root growth and is one of the most comprehensive root architectural models developed to date (Postma *et al.*, 2017). OpenSimRoot comprises a detailed 3-D model of root architecture and soil that accounts for interactions between root growth, construction costs, respiration and nutrient uptake at the scale of individual root segments over time. Because hypotheses focused on how individual components of root phenotypes interact with soil can be technically difficult to address through *in vivo* studies, simulation modelling can be a useful approach because it allows modification of individual phene states while other components of the plant phenotype and soil environment are held constant. As heuristic models, OpenSimRoot and its predecessor SimRoot (Lynch *et al.*, 1997) are useful tools to test the adequacy of our conceptual understanding of root–soil interactions, and have proven to be quite accurate for the prediction of fitness outcomes of root phenotypes under edaphic stress (e.g. Saengwilai *et al*., 2014, 2021; Galindo-Castañeda *et al*., 2018; Rangarajan *et al.*, 2018; Strock *et al*., 2018, 2021; Benes *et al.*, 2020; Perkins and Lynch, 2021).

We hypothesize that modifying the penetration ability of individual root classes will have important effects on the distribution of root length with depth, the carbon cost of the root system, the capture of resources such as nitrate, and plant fitness. To evaluate these hypotheses, we employ a new module for OpenSimRoot that accounts for interactions between soil physical properties, root growth and metabolic costs, allowing for the simulation of more realistic growth scenarios. We use this module to simulate growth of maize roots in three soil hardness regimes and investigate how altering the penetration ability of distinct root classes may have reciprocal effects on root length distribution and acquisition of mobile soil resources, using nitrate as the most important exemplar.

## MATERIALS AND METHODS

### Summary of OpenSimRoot

In OpenSimRoot, root system architecture is represented by a network of root nodes and is modelled as connected cylinders or truncated cones in three dimensions (Postma *et al.*, 2017). Root segment construction requires carbon and nutrients, and respiration and nutrient uptake are calculated at the root segment level. Branching angles, diameters and growth rates can be specified for each individual root class. The soil domain is simulated by a finite element model where each node contains values for water, nutrient content and soil physical properties such as bulk density. Shoot growth is simulated by abstract, non-geometric models and is represented by integral parameters such as leaf area from which light interception and photosynthesis are calculated. Carbon used for growth comes from either seed reserves or photosynthesis, and multiple components of metabolic costs include respiration, tissue nutrient content, nitrogen (N) fixation, nutrient uptake and production of exudates. Carbon is partitioned among roots, shoots and leaves according to potential growth rates limited by stress and available resources. Nutrient stresses have defined impacts on the rate of leaf area expansion, photosynthesis and root growth rates.

In OpenSimRoot, the sink strength of a given organ is based on the resource requirements for potential growth and maintenance of the tissue. Following Pages (2000), roots of greater thickness have greater longitudinal potential growth rates and, consequently, have greater sink strength. Resources required for root growth are determined by the volumetric increase associated with the class, location and age of each root segment. Respiration is a function of the root segment biomass and age.

Nutrient uptake by root segments follows Michaelis–Menten kinetics, the parameters of which can vary over time and by root class. The flow of nutrients to roots is simulated by convection–dispersion–diffusion equations, for which two implementations are available. (1) The Barber–Cushman model (Barber and Cushman, 1981) is high resolution, simulates nutrient depletion around roots and as such is suitable for immobile nutrients such as phosphorus. (2) An implementation of the solute model in SWMS3D (Šimunek *et al.*, 1995) models soil nutrient flows by coupling to the finite element soil water model. This is suitable for mobile nutrients such as nitrate. Organic matter mineralization follows the model of Yang and Janssen (2000). Ammoniacal N is not modelled, as nitrification is generally rapid in the field conditions being simulated (Barber, 1995).

OpenSimRoot functions by coupling various minimodels, each of which accounts for specific components (state variables) of plant growth and interactions with the environment. Minimodels integrate parameters of root growth, nutrient uptake and resource allocation, and are based on empirical measurements to model the relationship between root growth and soil resource acquisition (Postma *et al.*, 2017). A collection of minimodels related to a certain aspect of plant functioning, such as photosynthesis, respiration or root water uptake, is referred to as a module. Further information on OpenSimRoot is provided by Postma *et al.* (2017).

### Impedance module

In the soil impedance module of OpenSimRoot introduced and implemented in this study, soil strength is represented as impedance to linear penetration, in units of kiloPascals (kPa). The impedance then scales the linear extension and diameter growth rates of each root according to two user-specifiable functions, allowing flexible simulation of differing growth responses: (1) a soil strength function that relates soil strength to soil water status and soil bulk density and (2) a function to calculate the soil strength-dependent root impedance factor which defines the root elongation rate as limited by soil physical conditions (Supplementary data Fig. S1).

If soil strength is known (e.g. from penetrometer measurements), it may be specified directly as a constant, a depth profile or a 3-D field of any desired spatial resolution. In the case where penetration resistance is not explicitly known, OpenSimRoot computes it dynamically using the pedotransfer function of Gao *et al.* (2016) from bulk density, water status and position in the soil column [eqn (1)]. Soil porosity is estimated from bulk density if it is not provided, and all other inputs to the pedotransfer function are already available from OpenSimRoot’s existing water flow module. Whether impedance is specified as an input or computed from a pedotransfer function, the provided information is downscaled using OpenSimRoot’s existing interpolation routines (Postma *et al.*, 2017) to compute the impedance at the precise time and place where root growth occurs, thus allowing dynamic representation of the interactions between soil hardness and water content as they evolve through the growing period. Impedance at a point is assumed to be equal in all directions (i.e. it is a scalar not a tensor), and thus allows simulation of responses that change the rate but not the direction of root growth.


$$\begin{array}{l} Q=\rho {\left(A^{*}\frac{{\left(F-e\right)}^{2}}{1+e}{\left(\sigma_{s}^{\;p}-\;\psi S^{*}\right)}^{\;f}\right)}^{2} \end{array}$$ (1)

Equation (1) shows calculation of soil penetration resistance *Q* where *ρ* is soil bulk density, *e* is soil porosity, *σ*_s_ is net stress calculated from the weight of overlying soil layers, *ψ* is the soil matric potential calculated from water content and S* is the degree of saturation. A*, *F*, *p* and *f* are all fitted constants. (Gao *et al.*, 2016)

### Linking soil physical properties and root growth responses

**Fig. 1.**
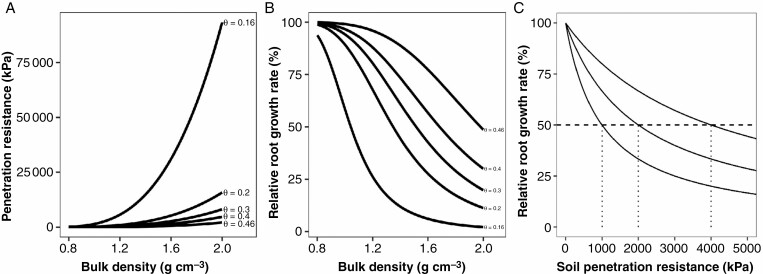
The relationship between penetration resistance and bulk density (A), the relative root growth rate and bulk density (B) and relative root growth rate and soil penetration resistance (C). Here we use a decreasing Michaelis–Menten with *K*_m_ = {1000, 2000, 4000}. The resulting effect of bulk density on relative growth rate (B) is roughly sigmoid, with a quasi-linear portion in the range typical of agricultural soils.


The relationship between soil penetration resistance and root elongation can be approximated by an impedance function that is unity at zero soil strength and decreases linearly to zero at a critical soil strength value at which root growth stops (Taylor and Gardner, 1963; Taylor *et al.*, 1966; Taylor and Ratliff, 1969; Voorhees *et al.*, 1975; Goss, 1977; Bengough and Mullins, 1990, 1991). The module represents this relationship between soil penetration resistance and root growth by reducing the elongation rate of each root as mechanical impedance increases, following a Michaelis–Menten-type saturating response [eqn (2)] that reaches 50 % slowdown in growth rate at a user-specified soil hardness (Fig. 1C).

$$\begin{array}{l} R_{I}=1-\frac{Q}{Q_{50}+Q}\;\;\; \end{array}$$ (2)

Equation (2) shows calculation of growth rate reduction *R*_I_ where *Q* is soil penetration resistance and *Q*_50_ is the *Q* at which rate reduction = 0.5.

The *Q*_50_ value in this function controls the degree of root response to impedance, with greater *Q*_50_ equating to less sensitivity of root growth rate in response to mechanical impedance (i.e. greater root penetration ability) (Fig. 1B). Under typical field conditions (bulk density in the range of 1.1 to 1.6 g cm^–3^ and soil water contents above the wilting point) the convolved output of the soil hardness and relative growth functions is close to linear, consistent with published observations of quasi-linear reductions in root growth rate as bulk density increases (Fig. 1).

The module also includes a root expansion term [eqn (3)] that scales the diameter of each root according to a user-specifiable value, allowing flexible simulation of differing growth responses. The root diameter consequently increases proportionally as elongation slows [eqn (3)]. This shift in root diameter is parameterized to be a response to a slowdown of root elongation and does not affect the penetration ability of the root. This root expansion term is not directly proportional to mechanical impedance and simply provides the interaction between mechanical impedance and root construction costs.


$$\begin{array}{l} D_{I}=\frac{1}{{R_{I}}^{\alpha }} \end{array}$$ (3)

Equation (3) shows calculation of root diameter increase *D*_I_, where *R*_I_ is growth rate reduction and *α* is a proportionality constant.

### Parameterizing soil bulk density profiles

To gain a systems-level understanding of how soil bulk density regimes may affect soil resource acquisition, three soil bulk density profiles representing common scenarios in agricultural systems were parameterized (Table 1; Supplementary data Fig. S2): uniform (1.0 g cm^–3^ throughout the profile), gradient (linear increase from 1.0 g cm^–3^ at the soil surface to 1.6 g cm^–3^ at 150 cm depth) and plow pan (1.6 g cm^–3^ from 20 to 30 cm, the same as gradient at all other depths). Soil parameters used for this model were taken from the Russell E. Larson Agricultural Research Farm at Rock Springs, PA (40.70°N, –77.95°W), which has a Hagerstown soil series (Hagerstown silt loam, fine, mixed, semi-active, mesic Typic Hapludalf) with 9.4 % sand, 67.1 % silt and 23.5 % clay.

**Table 1.** Bulk density was varied with depth to represent three soil scenarios

Profile	Bulk density (g cm^–3^)
Uniform	1 g cm^–3^ at all depths
Gradient	Linear increase in bulk density with depth from 1 g cm^–3^ at the surface to 1.6 g cm^–3^ at 150 cm from the surface
Plow pan	Linear increase in bulk density with depth from 1 g cm^–3^ at the surface to 1.6 g cm^–3^ at 150 cm from the surface; 1.6 g cm^–3^ from 20 to 30 cm from the soil surface

The van Genuchten parameters along with saturated hydraulic conductivity were estimated based on the soil composition and bulk density, and these were used to model water transport as well as nitrate transport through the soil (Supplementary data Table S1). Soil hydraulic properties were determined using the program ROSETTA (Schaap *et al.*, 2001), which estimates unsaturated hydraulic properties from surrogate soil data including soil texture and bulk density. The R function ‘ROSETTA’ from the soilDB package (Beaudette *et al.*, 2021) was used to determine hydraulic properties using sand, silt and clay percentages and bulk density as input parameters at each depth. The change in water content as determined by the water flow module further determines the soil strength in a spatio-temporally dynamic manner. Soil can lose water by evaporation and gain water by infiltration after precipitation, and affects nitrate movement in the simulated soil. Nitrate availability with time was modified by varying the precipitation levels as well as initial nitrate concentration such that three soil regimes had (1) high nitrate (322.5 kg ha^–1^) which leached through the soil quickly, (2) low nitrate (107.5 kg ha^–1^) which leached through the soil quickly and (3) high nitrate (322.5 kg ha^–1^) which had little leaching (Supplementary data Table S2). While the concentrations of nitrate were varied at the start of the simulation, the spatial distribution of nitrate was the same across these three treatments at the initial time step. By modifying the mobility of nitrate via the precipitation level, differences in the spatial distribution of nitrate accrued over time as the simulation progressed. Evaporation was kept at default values.

### Parameterizing root responses to penetration resistance

To investigate how variation in the penetration ability of different root classes may interact with these bulk density gradients to affect soil foraging strategies, four distinct combinations of root penetration abilities were parameterized by setting the growth-halving impedance (*Q*_50_) of axial and lateral root classes to either 1000 or 4000 kPa. The parameters corresponding to the different root phenotypes are presented in Table 2. In these simulations, the root expansion term was not varied and was kept constant at 0.4.

**Table 2.** Four distinct combinations of root penetration abilities were parameterized

Root phenotype	Axial–lateral	Parameterization
Weakly penetrating axials, weakly penetrating laterals	1000–1000	The relative elongation rate of both axial and lateral root growth was reduced by 50 % at 1000 kPa of penetration resistance (*Q*_50_ = 1000 kPa)
Weakly penetrating axials, strongly penetrating laterals	1000–4000	The relative elongation rate of axial root growth was reduced by 50 % at 1000 kPa of penetration resistance (*Q*_50Axial_ = 1000 kPa) while the relative elongation rate of laterals was reduced by 50 % at 4000 kPa of penetration resistance (*Q*_50Lateral_ = 4000 kPa)
Strongly penetrating axials, weakly penetrating laterals	4000–1000	The relative elongation rate of axial root growth was reduced by 50 % at 4000 kPa of penetration resistance (*Q*_50Axial_ = 4000 kPa) while the relative elongation rate of laterals was reduced by 50 % at 1000 kPa of penetration resistance (*Q*_50Lateral_ = 1000 kPa)
Strongly penetrating axials, strongly penetrating laterals	4000–4000	The relative elongation rate of both axial and lateral root growth was reduced by 50 % at 4000 kPa of penetration resistance (*Q*_50_ = 4000 kPa)

Plant growth of all four models was simulated for 40 d in each of the three soil bulk density profiles in the three different N regimes. All other plant growth and environmental parameters were held constant across all simulations. Six replicates of each model were run to account for the effects of stochasticity in the model. The simulated soil volume was 0.6 × 0.26 × 1.5 m deep to emulate a soil volume similar to that available to one plant within a planted row in the field. Roots that touched the vertical edges of the simulated area were reflected back in order to account for roots of neighbouring plants in a field environment and mimic the available soil volume of field conditions. The simulations reported here were performed using Git revision ac842ebfba423 of OpenSimRoot, and the current version including the impedance module is publicly available (https://gitlab.com/rootmodels/OpenSimRoot). All parameter files, run scripts and code needed to compile our version of OpenSimRoot are available at: https://doi.org/10.5281/zenodo.5649468

### Data analysis

Data were visualized using R version 3.6.2 (R Core Team, 2019). Root phenotypes were visualized with Paraview 5.5.2 (Ahrens *et al.*, 2005). Several OpenSimRoot model parameters are sampled from distributions using a random number generator, which causes stochasticity in model runs. Six replications with different random number generator seeds were performed for all treatments, and raw data are shown where appropriate to demonstrate when treatment effects are larger than those due to stochasticity. Performing statistical hypothesis testing on model results of this type is not appropriate for several reasons: (1) sample size and statistical power are somewhat arbitrary in simulation modelling, which increases the risk of identifying differences that are statistically but not biologically significant; (2) knowledge of the model makes the null hypothesis false *a priori*, rendering the hypothesis framework inappropriate’ and (3) structural–functional models make certain simplifying assumptions out of necessity, which could mean that variance present in simulation results is different from what would be observed *in vivo* (White *et al.*, 2014). Consequently, comparisons of model outputs need to be interpreted within the context of simulation assumptions. Additionally, because validation data for whole root system responses to soil physical conditions are scarce, the impedance module prioritizes flexibility and simplicity over physical parameter interpretations. Consequently, outputs from the impedance module should be treated as hypotheses and not predictions.

## RESULTS

### Soil physical parameters affect the distribution of root length

**Fig. 2.**
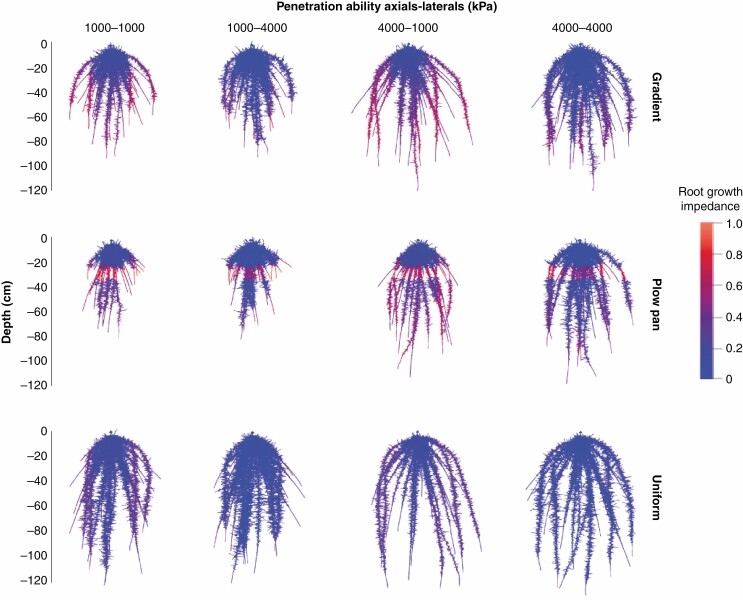
Root architecture and length per soil layer of 40-day-old maize root systems with variable penetration ability of axial and lateral root classes [*Q*_50_ = 1000 kPa (weakly penetrating) and *Q*_50_ = 4000 kPa (strongly penetrating)]. Roots are colourized by root growth impedance. Growth of these root systems was simulated in soils with bulk density profiles classified as uniform (1.0 g cm^–3^), severe plow pan (1.6 g cm^–3^ from 20 to 30 cm, within a gradient of 1.0 g cm^–3^ at the surface to 1.6 g cm^–3^ at 150 cm) or increasing with depth (1.0 g cm^–3^ at the surface to 1.6 g cm^–3^ at 150 cm).

The three distinct soil bulk density profiles had dramatic effects on the net length and distribution of root systems in simulated maize plants (Fig. 2). While relative root growth rate was homogenous for all parts of the root system in the uniform bulk density regime, growth rate was suppressed in deep soil by the increasing bulk density of the gradient regime, and in the compressed 20–30 cm horizon of the plow pan regime (Fig. 2).

### Root penetration ability and soil bulk density affect metabolic costs of soil foraging

**Fig. 3.**
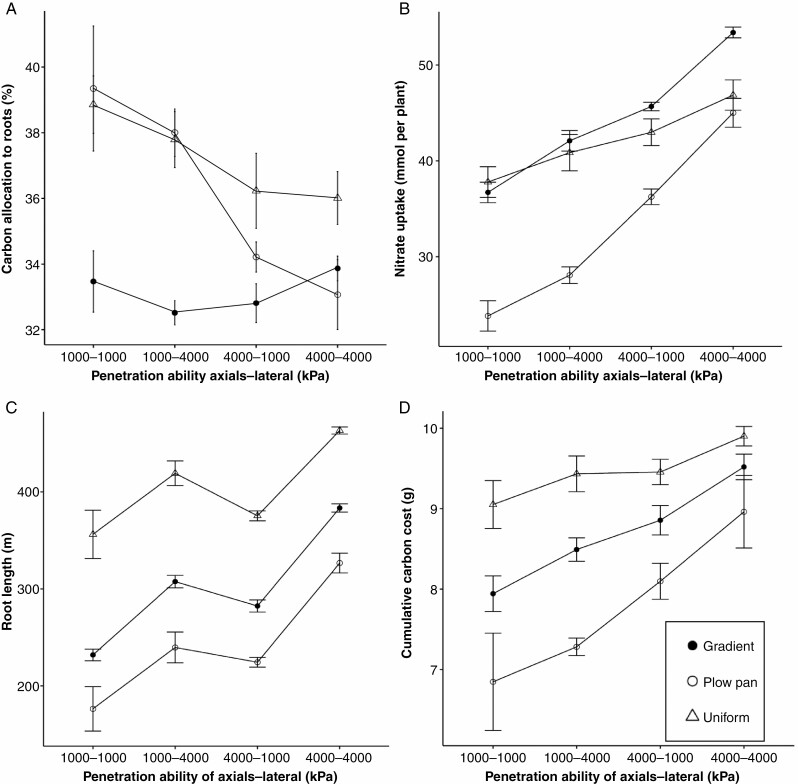
Percentage of total plant carbon allocated to the root system (A), nitrate uptake (B), total root system length (C) and cumulative carbon cost of 40-day-old maize root systems (D) with variable soil penetration ability by root class in soils with contrasting bulk density regimes. Data shown are from the high nitrate availability, high leaching environment. Soil bulk density was parameterized to be uniform (1.0 g cm^-3^), have the presence of a plow pan (1.6 g cm^–3^ from 20 to 30 cm, within a gradient of 1.0 g cm^–3^ at the surface to 1.6 g cm^–3^ at 150 cm) or have a gradient of increasing bulk density with depth (1.0 g cm^–3^ at the surface to 1.6 g cm^–3^ at 150 cm). Penetration ability of axial and lateral roots was modified between *Q*_50_ = 1000 kPa (weakly penetrating) and *Q*_*50*_ = 4000 kPa (strongly penetrating).

**Fig. 4.**
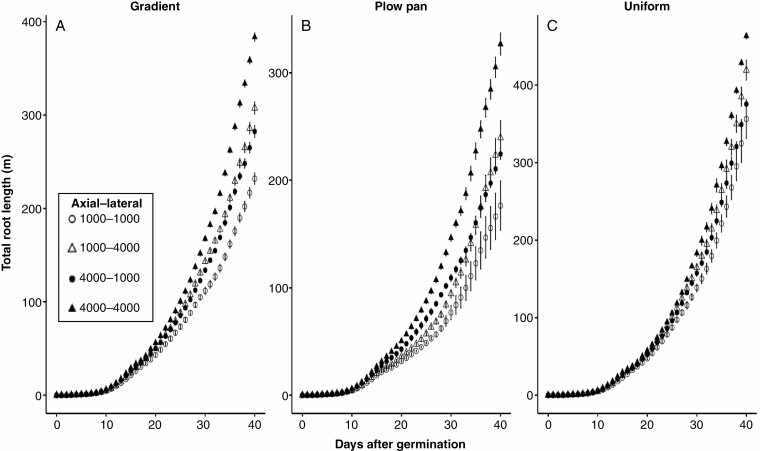
Total length from 0 to 40 d after germination for maize root systems with variable soil penetration ability by root class in soils with contrasting bulk density regimes. Data shown are from the high nitrate availability, high leaching environment. Soil bulk density was parameterized to be uniform (1.0 g cm^–3^), have the presence of a plow pan (1.6 g cm^–3^ from 20 to 30 cm, within a gradient of 1.0 g cm^–3^ at the surface to 1.6 g cm^–3^ at 150 cm) or have a gradient of increasing bulk density with depth (1.0 g cm^–3^ at the surface to 1.6 g cm^–3^ at 150 cm). Penetration ability of axial and lateral root classes was modified between *Q*_50_ = 1000 kPa (weakly penetrating) and *Q*_50_ = 4000 kPa (strongly penetrating).

In addition to modifying the relative growth rate of roots, bulk density profiles had distinct effects on the carbon cost of the root system (Fig. 3D). Compared with the uniform soil profile, net carbon costs of the root system were reduced (Fig. 3D) in the gradient and plow pan scenarios due to inhibition of root elongation (Fig. 3C). Root systems with improved penetration ability in these bulk density profiles had elevated root carbon costs (Fig. 3D), reflective of greater root length in these plants (Figs 3C and 4). No clear differences in total length or carbon cost were observed between root systems with enhanced penetration ability of either axial or lateral root classes alone (Fig. 3C, D and 4A, B). Due to the limited size of the root system, plants in the gradient and plow pan scenarios had proportionally less carbon allocated to their root systems than plants in the uniform bulk density scenario (Fig. 3A). Furthermore, compared with plants in the uniform scenario, plants in soils with a gradient of bulk density displayed greater shoot size relative to the cumulative carbon cost of the root system (Supplementary data Fig. S3). Scenarios with high leaching of nitrate further reduced the size of the shoot relative to the carbon allocated to the roots in soils with uniform and plow pan profiles (Supplementary data Fig. S3A, B)

### Penetration ability of distinct root classes affects rooting depth

**Fig. 5.**
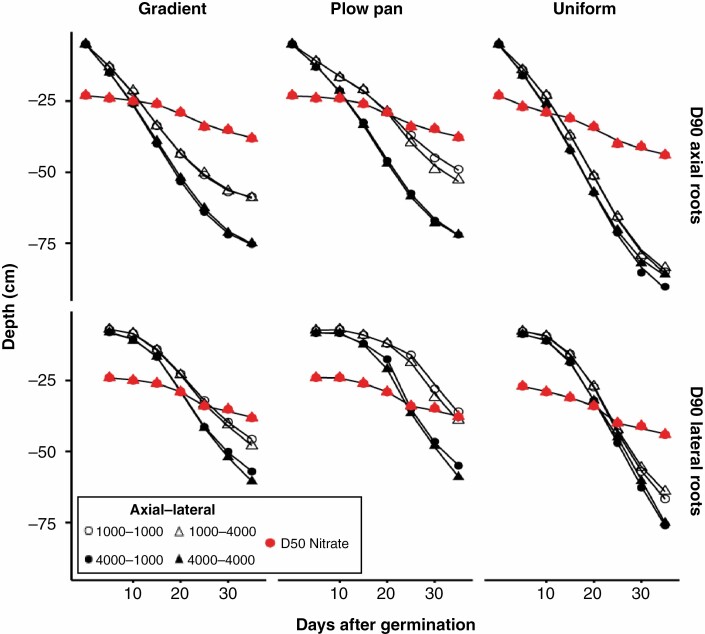
Depth of 90 % of the root length in lateral and axial classes (D90 roots) and the depth of 50 % of the available nitrate (D50 nitrate) over time for maize root systems with variable soil penetration ability in soils with contrasting bulk density regimes. Data shown are from the high nitrate availability, high leaching environment. Soil bulk density was parameterized to be uniform (1.0 g cm^–3^), have the presence of a plow pan (1.6 g cm^–3^ from 20 to 30 cm, within a gradient of 1.0 g cm^–3^ at the surface to 1.6 g cm^–3^ at 150 cm) or have a gradient of increasing bulk density with depth (1.0 g cm^–3^ at the surface to 1.6 g cm^–3^ at 150 cm). Penetration ability of axial and lateral root classes was modified between *Q*_50_ = 1000 kPa (weakly penetrating) and *Q*_*50*_ = 4000 kPa (strongly penetrating).

**Fig. 6.**
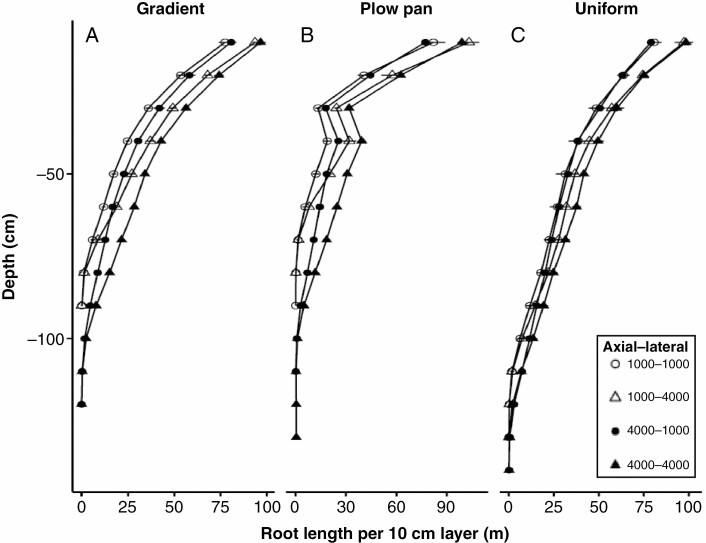
Distribution of root length at 40 d after germination in maize root systems with variable penetration ability of different root classes in soils with contrasting bulk density regimes. Data shown are from the high nitrate availability, high leaching environment. Soil bulk density was parameterized to be uniform (1.0 g cm^–3^), have the presence of a plow pan (1.6 g cm^–3^ from 20 to 30 cm, within a gradient of 1.0 g cm^–3^ at the surface to 1.6 g cm^–3^ at 150 cm) or have a gradient of increasing bulk density with depth (1.0 g cm^–3^ at the surface to 1.6 g cm^–3^ at 150 cm). Penetration ability of axial and lateral root classes was modified between *Q*_50_ = 1000 kPa (weakly penetrating) and *Q*_50_ = 4000 kPa (strongly penetrating).

Adjustments in the sensitivity of root elongation to mechanical impedance had clear effects on relative growth rates in soils with high bulk densities, which in turn affected the distribution of root length across depth (Figs 2, 5 and 6). The effect of increasing penetration ability on total root length and depth was most evident in soils with a plow pan or gradient bulk density profile (Figs 4 and 5). Altering the penetration ability of different root classes (axial vs. lateral roots) also had contrasting effects on root length distribution. While enhancing the penetration ability of either axial or lateral roots produced comparably sized root systems (Figs 3C and 4), improving the penetration ability of axial roots produced greater distribution of root length in deeper regions of the soil profile, especially in soils with a plow pan or gradient of bulk density (Fig. 5). By 40 d after germination, root systems with strongly penetrating lateral roots had greater total root length in shallow regions of the soil profile, while those with strongly penetrating axial roots achieved greater maximum root depth and had greater total root length beyond 75 cm of soil depth (Fig. 6).

### Enhanced penetration ability and rooting depth improve nitrate capture

**Fig. 7.**
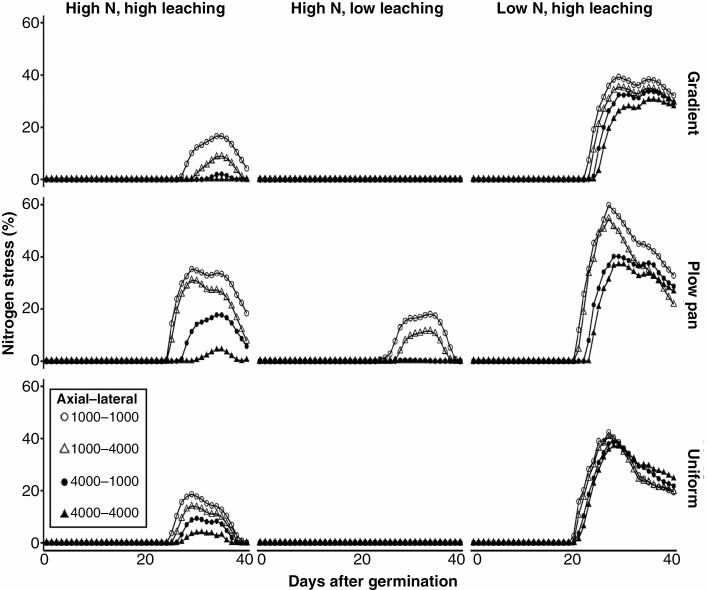
Nitrate stress from 0 to 40 d after germination in maize plants with variable penetration ability of different root classes in soils with contrasting bulk density regimes, initial nitrate availability and leaching. Nutrient stress is given as a percentage, which is calculated as [1 – (*u*–*m*)/(*o*–*m*)] × 100, where *u* is the quantity of nutrient acquired (nitrate), *o* is the optimal nutrient required by the plant and *m* is the minimal nutrient content required by the plant. 0 % indicates no stress, 100 % indicates severe stress. Soil bulk density was parameterized to be uniform (1.0 g cm^–3^), have the presence of a plow pan (1.6 g cm^–3^ from 20 to 30 cm, within a gradient of 1.0 g cm^–3^ at the surface to 1.6 g cm^–3^ at 150 cm) or have a gradient of increasing bulk density with depth (1.0 g cm^–3^ at the surface to 1.6 g cm^–3^ at 150 cm). Penetration ability of axial and lateral root classes was modified between *Q*_50_ = 1000 kPa (weakly penetrating) and *Q*_50_ = 4000 kPa (strongly penetrating).

The inhibition of rooting depth in soils with a plow pan or gradient of bulk density had a strong effect on nitrate capture, resulting in higher N stress beginning around 20–25 d after germination (Fig. 7). This increase in N stress was especially pronounced in soils with initially low availability or high leaching of nitrate (Fig. 7). Plants with improved penetration ability of axial roots and deeper distribution of root length had improved nitrate capture (Fig. 3B; Supplementary data Fig. S5) and reduced N stress (Fig. 7) compared with plants with improved penetration ability of lateral roots. This benefit of strongly penetrating axial roots for nitrate capture was especially pronounced in soils that had initially high nitrate, high leaching and plow pan or gradient of bulk density (Figs 3B and 7; Supplementary data Fig. S5).

**Fig. 8.**
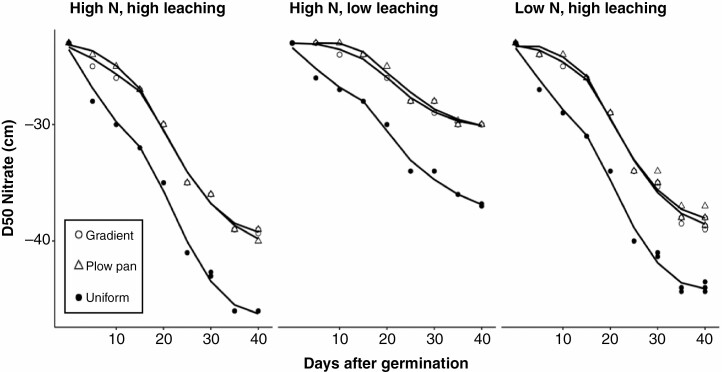
Depth of 50 % of the available nitrate in the soil from 0 to 40 d after germination in soils with contrasting bulk density regimes, initial nitrate availability and leaching. Soil bulk density was parameterized to be uniform (1.0 g cm^–3^), have the presence of a plow pan (1.6 g cm^–3^ from 20 to 30 cm, within a gradient of 1.0 g cm^–3^ at the surface to 1.6 g cm^–3^ at 150 cm) or have a gradient of increasing bulk density with depth (1.0 g cm^–3^ at the surface to 1.6 g cm^–3^ at 150 cm).

In addition to affecting the distribution of root length, soil bulk density profiles also had a strong effect on the availability and mobility of nitrate within the soil profile. While all scenarios were parameterized to have the same spatial distribution of nitrate at the initial time step, as the simulation progressed soils with a plow pan or gradient of bulk density had reduced leaching of nitrate from shallow domains of the soil profile compared with soils with a uniform bulk density (Fig. 8; Supplementary data Fig. S4). Due to the inhibition of root elongation at depth and reduced leaching of nitrate, soils with the gradient bulk density profile had greater spatio-temporal coincidence of root length distribution and nitrate availability (Fig. 5). While the total N uptake between plants in the gradient and uniform bulk density profiles was similar (Fig. 3B; Supplementary data Fig. S5), soils with a uniform profile of low bulk density had larger root systems (Figs 3C, 4 and 6), and the nitrate uptake per unit length of root in these scenarios was the smallest (Supplementary data Fig. S6). Consequently, the quantity of nitrate acquired per unit of carbon expended for soil resource extraction was greater in soils with a gradient of bulk density compared with those with a uniform, unimpeded profile (Supplementary data Fig. S7).

**Fig. 9.**
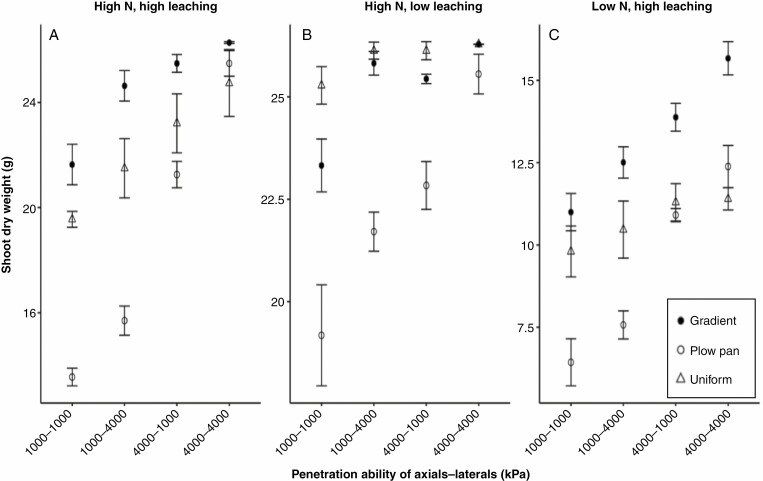
Shoot dry weight of 40-day-old maize plants with variable penetration ability of different root classes in soils with contrasting bulk density regimes, initial nitrate availability and leaching. Soil bulk density was parameterized to be uniform (1.0 g cm^–3^), have the presence of a plow pan (1.6 g cm^–3^ from 20 to 30 cm, within a gradient of 1.0 g cm^–3^ at the surface to 1.6 g cm^–3^ at 150 cm) or have a gradient of increasing bulk density with depth (1.0 g cm^–3^ at the surface to 1.6 g cm^–3^ at 150 cm). Penetration ability of axial and lateral root classes was modified between *Q*_50_ = 1000 kPa (weakly penetrating) and *Q*_50_ = 4000 kPa (strongly penetrating).

Plant dry weight at 40 d after germination highlights the strong effect of soil density profiles on overall plant growth (Fig. 9), where the gradient profile produced the largest shoots of all three scenarios (Fig. 9). This larger shoot size in the gradient bulk density profile was the result of reduced allocation of carbon resources to the root system (Fig. 3A, D), as well as reduced leaching of nitrate in the gradient scenario (Fig. 8; Supplementary data Fig. S4), thereby improving the balance of resources devoted to soil foraging and nitrate acquisition compared with the soils with uniform bulk density (Supplementary data Figs S3, S5 and S6). Additionally, there was an interaction between the soil bulk density profiles and root penetration ability reflected in these shoot mass data, where the value of strongly penetrating axial roots for improved plant growth is greater under plow pan regimes than environments with a gradient of bulk density (Fig. 9). Furthermore, the fitness benefit of modifying root penetration ability is affected by availability and mobility of nitrate; where the effect of improved penetration ability of both axial and lateral roots on shoot dry weight is greater in soils where nitrate leaches readily (Fig. 9A, C).

## DISCUSSION

In this research, we consider how the heterogeneity of bulk density regimes of real-world soils affects root growth and soil resource acquisition. Functional–structural modelling provides a useful approach for addressing complex hypotheses regarding the broader interactions between soil physics, root growth and nutrient capture. Our results suggest that simple modifications in the sensitivity of root elongation to soil hardness are sufficient to alter how soil physical properties affect root length distribution (Figs 2, 5 and 6) and the metabolic cost of soil exploration (Fig. 3). Consequently, soil bulk density profiles and the penetration ability of distinct root classes have interacting effects on the acquisition of mobile resources such as nitrate (Figs 3, 5 and 7), thereby influencing plant performance (Fig. 9). Overall, these results highlight how heterogeneity in soil physical properties plays a significant role in determining the spatial distribution of the root system and the plant’s capacity to acquire soil resources.

Generally, soil N exists in three forms: (1) organic N compounds; (2) ammonium (NH_4_^+^); and (3) nitrate (NO_3_^−^). In most agroecosystems, ammonium is rapidly converted to nitrate, which is the dominant form of N acquired by crop plants (Barber and Cushman, 1981). In high-input agroecosystems where N fertilizer is intensively utilized, <50 % of applied N is captured by roots, while the balance is leached with irrigation or rainfall events and localized in deeper horizons over time, or volatilized (Dathe *et al.*, 2016; Thorup-Kristensen and Kirkegaard, 2016). We observe that nitrate foraging in deep soil domains under various compaction regimes is strongly affected by variation in the sensitivity of root elongation to mechanical impedance. While enhancing the penetration capacity of either axial or lateral roots increased the overall size of the root system in all bulk density regimes (Figs 3 and 4), the distribution of root length by depth was most strongly influenced by the elongation rate of the main root axes (Figs 5 and 6), similar to observations in modelling studies of other cereal crops (Bingham and Wu, 2011). As nitrate leached over time (Fig. 7), axial roots with greater penetration ability were capable of deeper soil exploration in soils with a plow pan or bulk density gradient (Figs 5 and 6), thereby increasing nitrate capture (Fig. 7; Supplementary data Fig. S7) and plant biomass (Fig. 9).

These observations align with the hypothesized advantages of the ‘steep, deep, cheap’ ideotype for improved N capture proposed by Lynch (2013), where exploration of deeper soil domains improves the acquisition of mobile resources such as nitrate. The fitness benefits of this foraging strategy have also been demonstrated *in vivo* where maize genotypes that produced few, thick nodal roots had a deeper distribution of root length and greater shoot growth under N deficit compared with genotypes that produced many, thin nodal roots (Schneider *et al.*, 2021*b*). Compaction can lead to decreased, constant or even increased root numbers (Bushamuka and Zobel, 1998; Grzesiak *et al.*, 2014; Colombi and Walter, 2016). However, a recent study reported that rooting depth in compacted soil is independent of the number of roots formed (Vanhees *et al.*, 2021). While the number of roots may not matter, our results show that the penetration ability of different root classes could be important in determining root length distribution as well as rooting depth. This is especially relevant to maize where different root classes and ages have different diameters, carbon costs and elongation rates (York and Lynch, 2015). The difference in penetration ability along with the other root class-specific characteristics ultimately affects the volume and depth of soil that can be explored within a specified time by different root classes. In addition to improving nitrate capture, axial roots capable of penetrating strong soils and promoting deeper soil exploration would probably increase plant water uptake under many drought scenarios, in which water is more available in deeper soil domains (Lynch, 2018). Water stress is common in non-irrigated agricultural systems, and shallow soil horizons are the first to dry as drought progresses, leading to superior water uptake associated with root systems with greater rooting depth (Lynch, 2013).

Enhanced penetration ability of either root class resulted in larger root systems that explored more soil volume (Figs 2–4), but this comes at an increased cost of producing and maintaining root length (Fig. 3). Roots are composed of heterotrophic tissues, and metabolic costs of the root system can be significant, consuming >50 % of the daily net carbon assimilated by the shoot (Nielsen *et al.*, 1998; Fan *et al.*, 2003). In low fertility environments, plants allocate an even greater fraction of their photosynthate to the root system (Van Der Werf *et al.*, 1988; Lambers *et al.*, 1996; Nielsen *et al*., 1998, 2001), thereby reducing growth of photosynthetic tissues. The net metabolic cost of the root system consists of three main components: (1) construction of new root tissue; (2) respiratory maintenance of existing root tissue; and (3) ion uptake (Van Der Werf *et al.*, 1988). Of these, root respiration allocated to maintenance can account for up to 90 % of total root respiration under nutrient limitation (Nielsen *et al.*, 1998). Modelling studies have similarly estimated that under N stress, root metabolic costs can contribute up to 40 % of the plant growth reduction (Postma and Lynch, 2011). Similarly, the increased metabolic burden of producing and maintaining root length in compacted soils limits available resources for greater root elongation, thereby hindering soil exploration, nitrate capture and overall plant growth. When broken down by root class, increasing the penetration ability of lateral roots increases root system size, but this added root length is distributed in the epipedon (Figs 3, 5 and 6). In contrast, increasing the penetration ability of axial roots resulted in similarly sized root systems, but with deeper distribution of root length (Figs 3, 5 and 6). Although root systems with a high penetration ability of both axial and lateral root classes had the largest root systems (Figs 3 and 4), there was minimal difference in rooting depth (Figs 5 and 6), N deficiency (Fig. 8) and shoot size (Fig. 9), but greater root carbon cost (Fig. 3) compared with plants with enhanced penetration of axial roots alone. These observations highlight the minimal benefit of enhancing the penetration ability of lateral roots for N capture in compacted soils, similar to other reports where maize root systems with dense lateral branching had increased competition among root axes for internal (e.g. carbohydrates) and external (e.g. nitrate) resources in N-deficient soils (Postma *et al.*, 2014; Zhan and Lynch, 2015; Rangarajan *et al.*, 2018).

Not only do plow pans and bulk density gradients reduce the overall size and depth of the root system (Figs 3–6), but soils of high bulk density also had less nitrate leaching (Fig. 7). The reduced pore space and connectivity in these soils diminishes soil hydraulic conductivity, thereby restricting water infiltration and mass flow of nutrients (Richard *et al.*, 2001; Lipiec and Hatano, 2003; Romero *et al.*, 2011). Consequently, in soils with a plow pan or bulk density gradient, nitrate as well as root length are confined to shallower soil domains. In soils parameterized to have a high rate of leaching, this co-localization of root length and nitrate resulted in larger plants by 40 d in the gradient profile compared with the uniform profile (Fig. 9A, C). This suggests that despite inhibiting root elongation, deeper soil strata with high bulk density may improve the utilization efficiency of applied N fertilizer by reducing the leaching of this resource. In scenarios where growth-limiting resources are heterogeneously distributed, this confinement of root growth to soil domains where the balance between resource expenditure and acquisition is improved appears to have significant effects on the efficiency of resource acquisition and overall plant performance. Nevertheless, this trend would probably only be applicable to a narrow range of environments where the benefit of inhibiting the infiltration of water and soluble nutrients outweighs the cost of limiting root growth. For example, in heavier textured soils where leaching of nitrate is less relevant to the optimization of plant fitness, the limitation to root elongation by compaction would probably be more of a detriment to soil exploration and resource acquisition than the benefit of reducing leaching. In contrast, in sandy soils, sub-soil compaction may not significantly interfere with the infiltration of water and nutrients into deep layers but can prevent roots from reaching and utilizing these resources (Laker, 2001). Additionally, it should be noted that while some soils, such as some Andosols or Histosols, may have only slight increases in bulk density with depth within the root zone, the parameterization of a uniform bulk density profile as we used in the present study would not be likely to be observed *in situ* and serves simply as a control for experimentation.

The concepts addressed in this research can be difficult or impossible to study empirically. Attempts to evaluate such questions in the field are often technically complicated, laborious and may be subject to artefacts. Heuristic models complement these shortcomings of *in vivo* research by testing the adequacy of hypotheses and identifying knowledge gaps prior to investing resources in field studies. Furthermore, output from modelling can have utility for sensitivity analysis to determine what phenotypes have the greatest impact on root system growth and resource capture (Bingham and Wu, 2011). These sensitivity analyses may subsequently be used to provide direction for more targeted physiological and genetic studies that hold the greatest promise (Dunbabin, 2007; Benes *et al.*, 2020). While we focused on variation in bulk density with depth in the present study, we did not consider the effects of variation in soil bulk density over time or in the horizontal dimension. Future work could explore more detailed soil structures (macropores, wheel-track compaction, etc.) as well as feedbacks over time (e.g. simulating the loosening of compacted layers by the roots of cover crops).

Another point worth noting is that OpenSimRoot treats lateral roots as being constitutively determinant, where loss of the apical meristem is part of the natural developmental sequence as has been observed *in vivo* (Shishkova *et al.*, 2008). The default parameterization of determinacy reflects observations of first-order lateral roots in field-grown maize (Varney and McCully, 1991). The model also assumes that the distance between the emergence sites of lateral roots is constant. This lateral root determinacy and fixed distance between emergence sites is a simplification of reality, where mechanical impedance to the elongation of parent root axes may encourage the elongation and continued emergence of lateral root primordia in non-compacted soil domains (Bingham and Bengough, 2003). While the general effects of impedance on root length distribution and overall plant growth are evident in this basic investigation, introducing plasticity of lateral root determinacy and emergence would probably result in amplification of these patterns through compensatory growth of the root system in non-compacted regions.

There is a need for 3-D root architectural models to simulate the dynamic development of root structure and its interaction with soil properties while taking into consideration the effects of carbon availability and allocation. By including an impedance module in OpenSimRoot, we provide a means of studying the effects of soil strength on root growth and architecture dynamically while accounting for carbon balances and soil resource capture. In general, crop simulation models that utilize detailed soil process sub-models require the incorporation of a large number of parameters (Wu *et al.*, 2007). Root impedance has been previously implemented in SPACSYS (Wu *et al.*, 2007), R-SWMS (Clausnitzer and Hopmans, 1994; Javaux *et al.*, 2008), HYDRUS (Hartmann *et al.*, 2018), RootTyp (Pages *et al.*, 2004), Rootmap (Diggle, 1988) and RootBox (de Moraes *et al*., 2018, 2019). In some of these models, however, the degree of mechanical impedance to root growth is determined from empirical relationships between bulk density, soil texture and water content (Jones *et al.*, 1991), requiring the determination of the effect of the relationship between these three factors on mechanical impedance for each soil. RootBox requires soil- and depth-specific empirical parameters to tune the relationship between water content and penetration resistance, while OpenSimRoot is broadly applicable with only the hydraulic parameters and includes an automatic adjustment for net stress as depth increases. Furthermore, many of these models only consider root growth at the scale of soil layers, and do not consider individual root classes as OpenSimRoot does. This is important as properties that facilitate root penetration ability may be distinct to certain root classes. For example, Schneider *et al.* (2021*a*) shows that multiseriate cortical sclerenchyma that improve the penetration ability of roots are only present in mature nodal roots of cereal crops. Similarly, genetic variation for root anatomical phenotypes in maize that affect root penetration ability are node specific (Yang *et al.*, 2019; Vanhees *et al.*, 2020). OpenSimRoot is also unique in that it permits the independent parameterization of bulk density and hydraulic parameters (i.e. conductivity and water storage capacity), allowing for the opportunity to explore the relative contributions of each factor to the growth of the root system.

### Conclusions

In this study, we demonstrate that soil bulk density profiles and the penetration ability of distinct root classes have interacting effects on acquisition of mobile resources such as nitrate, thereby influencing plant performance. Plow pans and bulk density gradients affected overall size, distribution and carbon costs of the root system. Soils with high bulk density at depth impeded rooting depth and reduced leaching of nitrate, thereby improving the coincidence of N and root length. While increasing penetration ability of either axial or lateral root classes produced root systems of comparable net length, improved penetration of axial roots increased allocation of root length in deeper domains, thereby amplifying N acquisition and shoot biomass. Although enhanced penetration ability of both root classes was associated with greater root system carbon costs, the benefit to plant fitness from improved soil exploration and resource capture offset these. While lateral roots comprise the bulk of root system length, axial roots function as a scaffold determining the distribution of these laterals. In soils with high soil strength and leaching, root systems with enhanced penetration ability of axial roots have greater distribution of root length at depth, thereby improving capture of mobile resources. Although the complexities of interaction between root growth and soil physical properties are difficult to investigate through *in vivo* experimentation, the use of functional–structural plant models can successfully integrate processes relevant to root growth and soil physics that are difficult to measure empirically. We envisage that models such as OpenSimRoot will be used and expanded by the plant and soil science communities to simulate root system growth and nutrient and water uptake in an ever-widening scenario for species, environments and crop management practices to advance root-based opportunities that increase the sustainability of agricultural systems.

## SUPPLEMENTARY DATA

Supplementary data are available online at https://academic.oup.com/aob and consist of the following.

Table S1: Bulk density and the van Ganuchten parameters. 

Table S2: OpenSimRoot parameterization for nitrogen and leaching regimes.

Fig. S1: Conceptual diagram of factors affecting root growth rate in OpenSimRoot.

Fig. S2: Visualization of bulk density across depth in these three bulk density regimes.

Fig. S3: The ratio of shoot dry weight to cumulative carbon cost of the root system in 40-day-old maize plants with variable penetration ability of different root classes in soils with contrasting bulk density regimes, initial nitrate availability and leaching.

Fig. S4: Distribution of nitrate by depth at 20 and 35 d after germination in soils with contrasting bulk density regimes, initial nitrate availability and leaching.

Fig. S5: Nitrate uptake over 40 d of growth in maize plants with variable penetration ability of different root classes in soils with contrasting bulk density regimes, initial nitrate availability and leaching.

Fig. S6: Nitrate uptake per metre of root from 0 to 40 d after germination in maize plants with variable penetration ability of different root classes in soils with contrasting bulk density regimes.

Fig. S7: Ratio of nitrate uptake to cumulative carbon allocated to the root system in 40-day-old maize plants with variable penetration ability of different root classes in soils with contrasting bulk density regimes.

mcab144_suppl_Supplementary_Figures

mcab144_suppl_Supplementary_Tables
