# Supplementary figures and images for: Theoretical evidence that root penetration ability interacts with soil compaction regimes to affect nitrate capture
Source: Ann Bot. 2021 Nov 30;129(3):315–30. doi: 10.1093/aob/mcab144 (PMC8835659; doi:10.1093/aob/mcab144)

**SUPPLEMENTARY FIGURES**


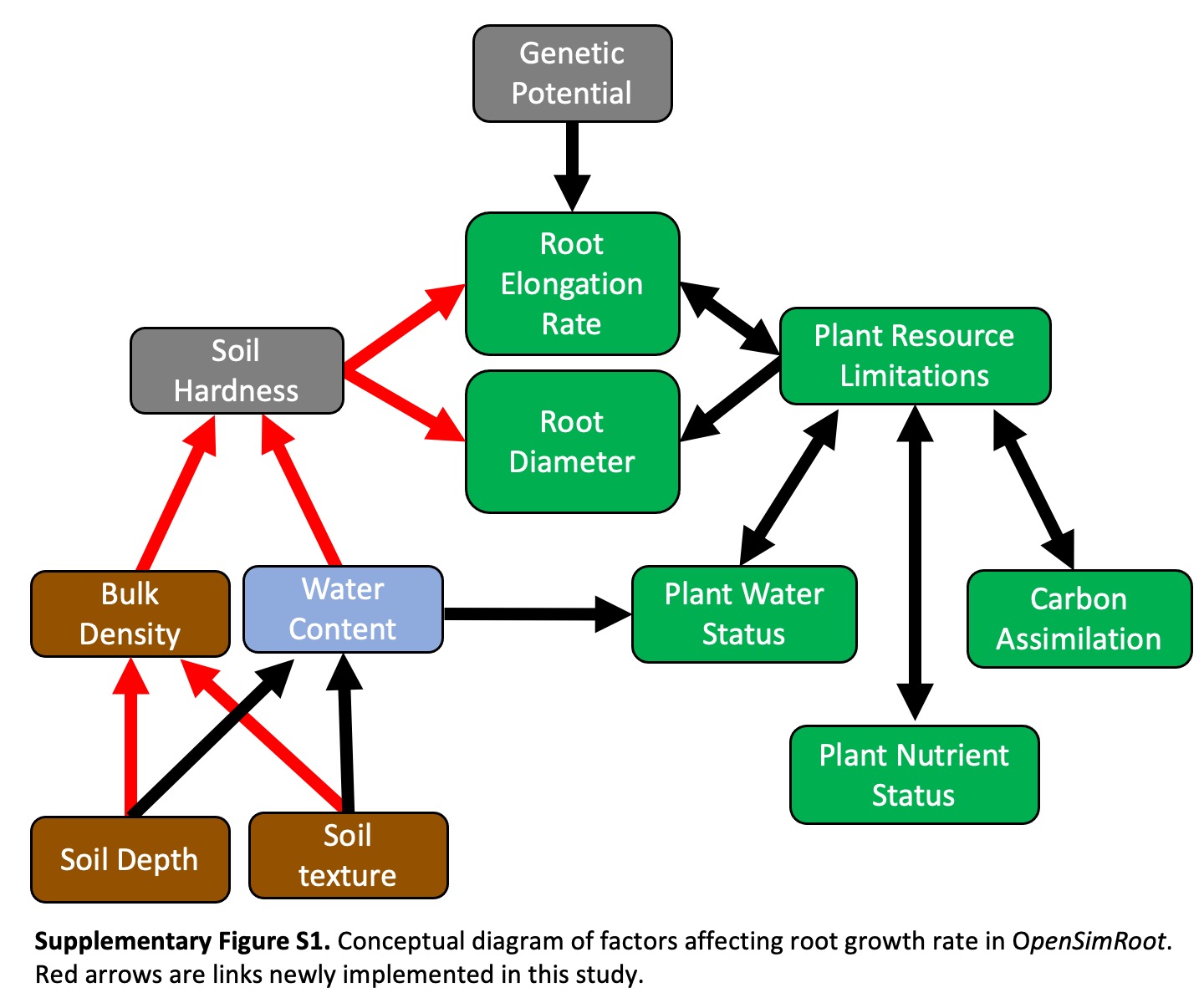

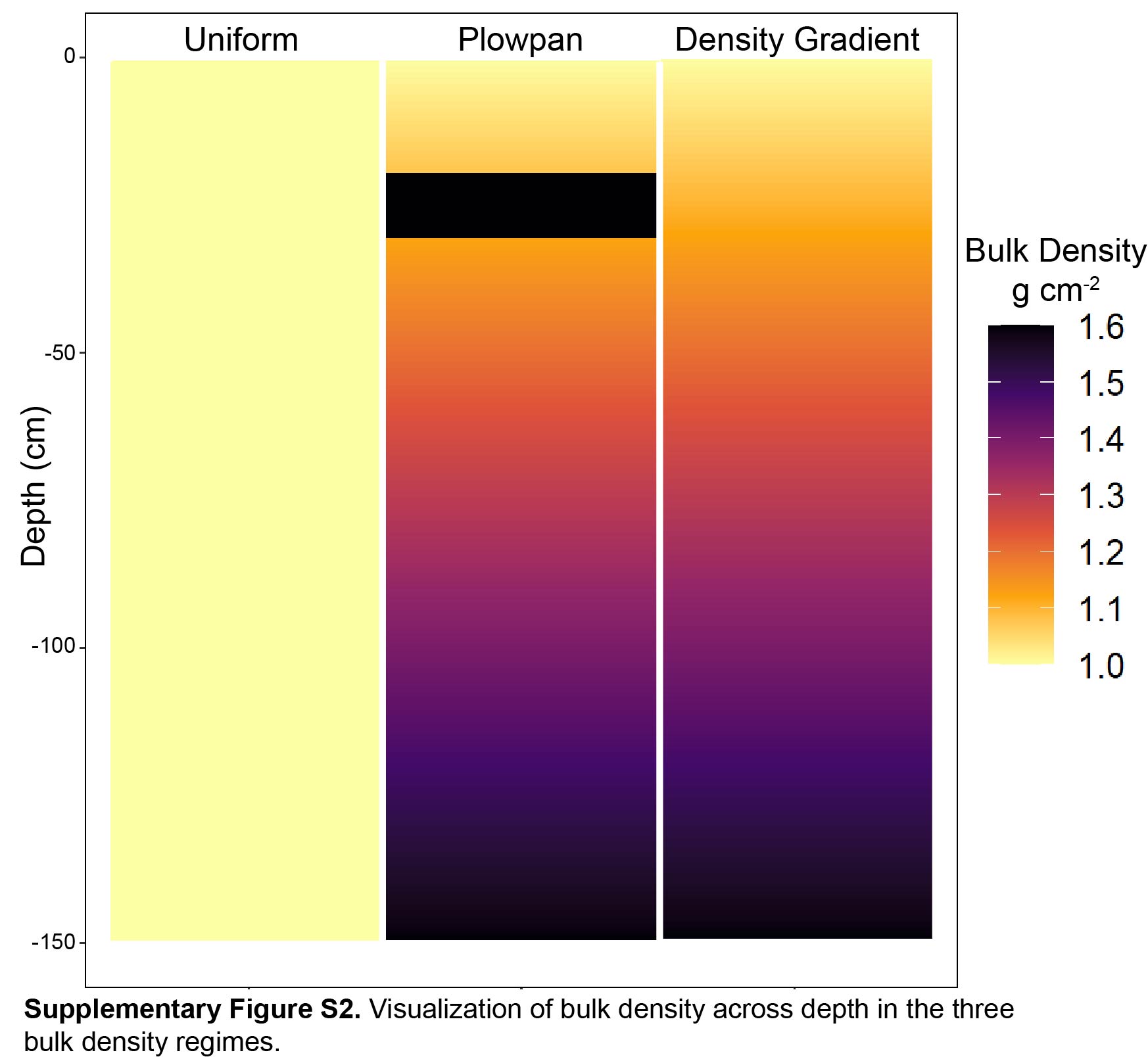

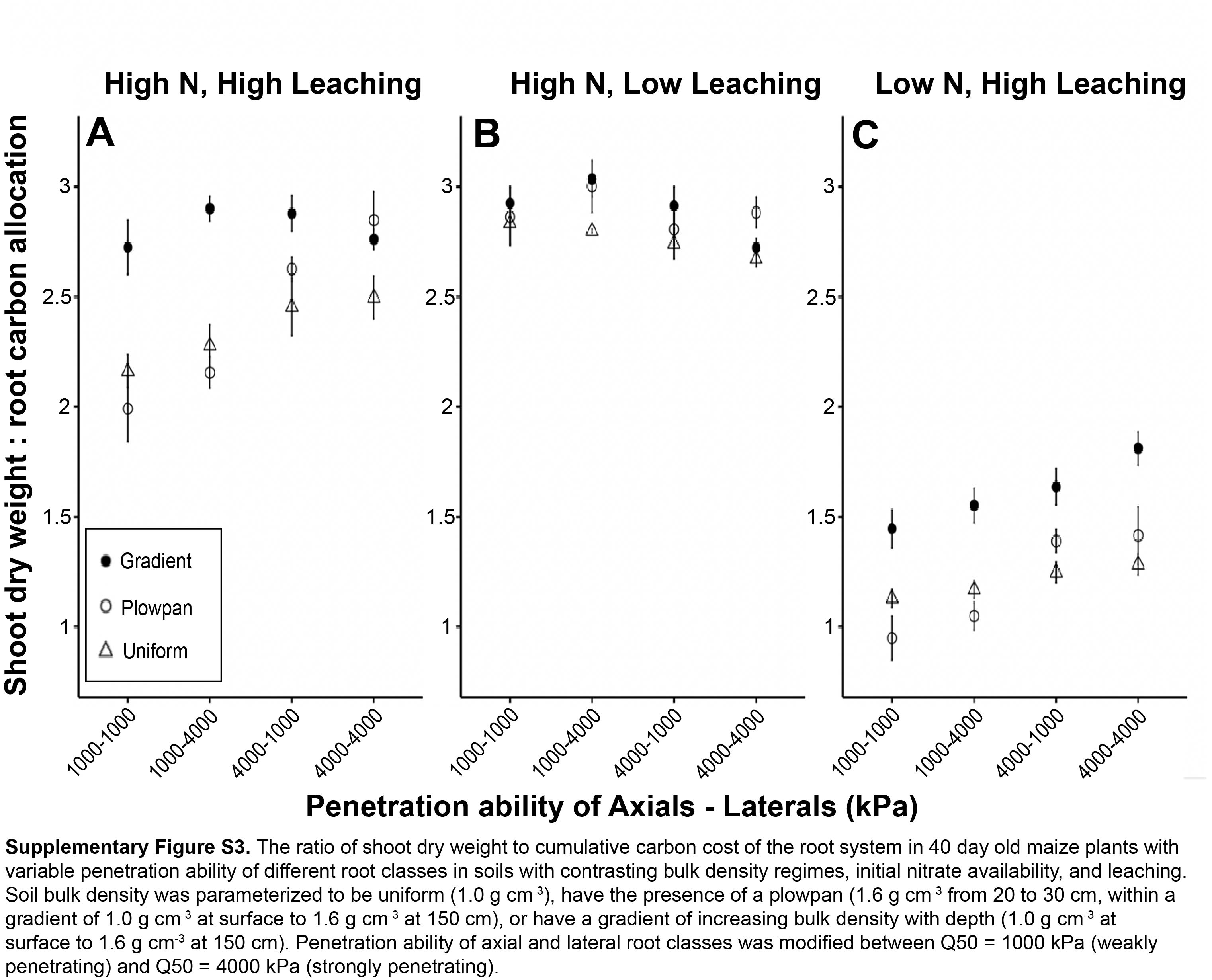

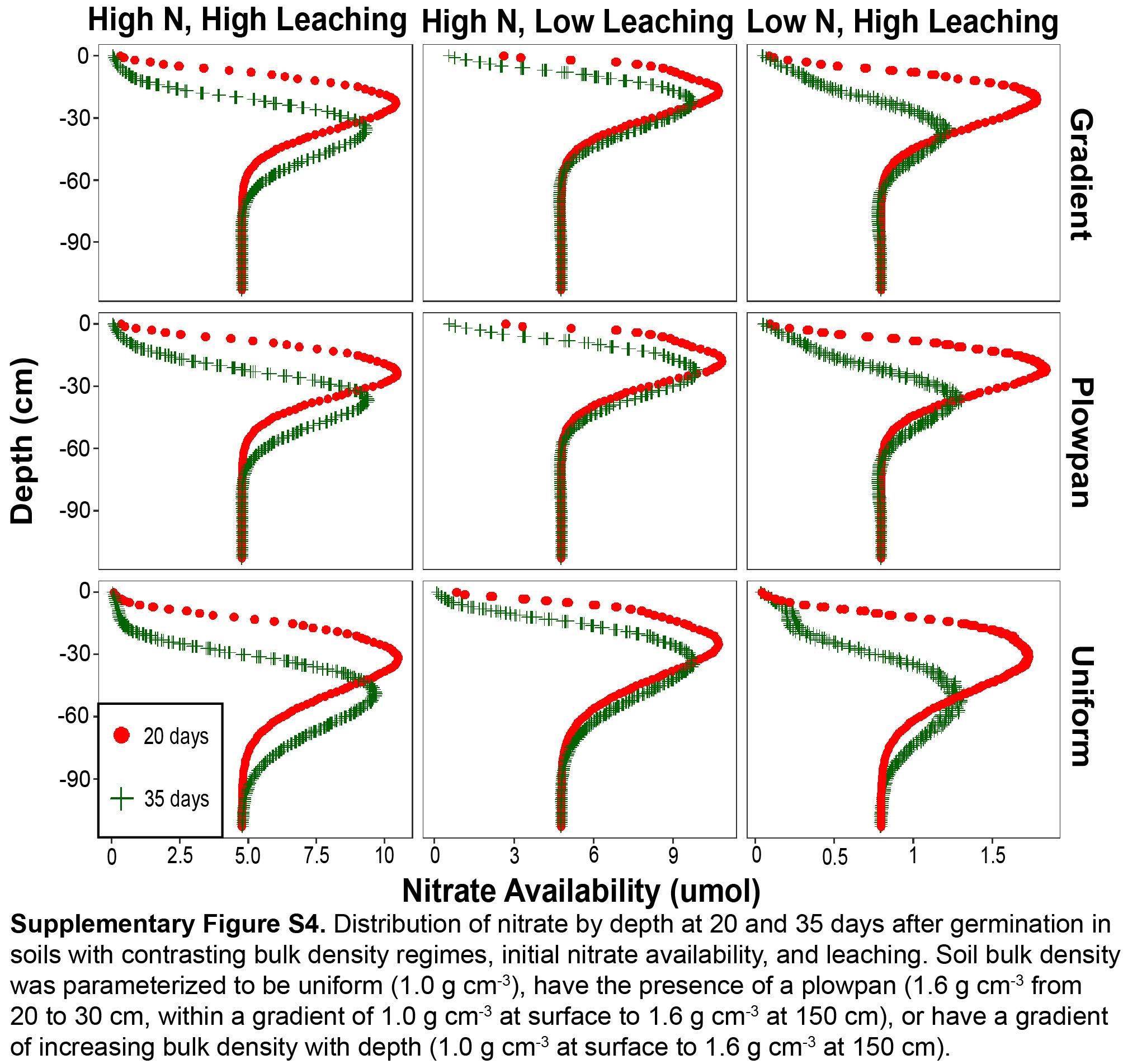

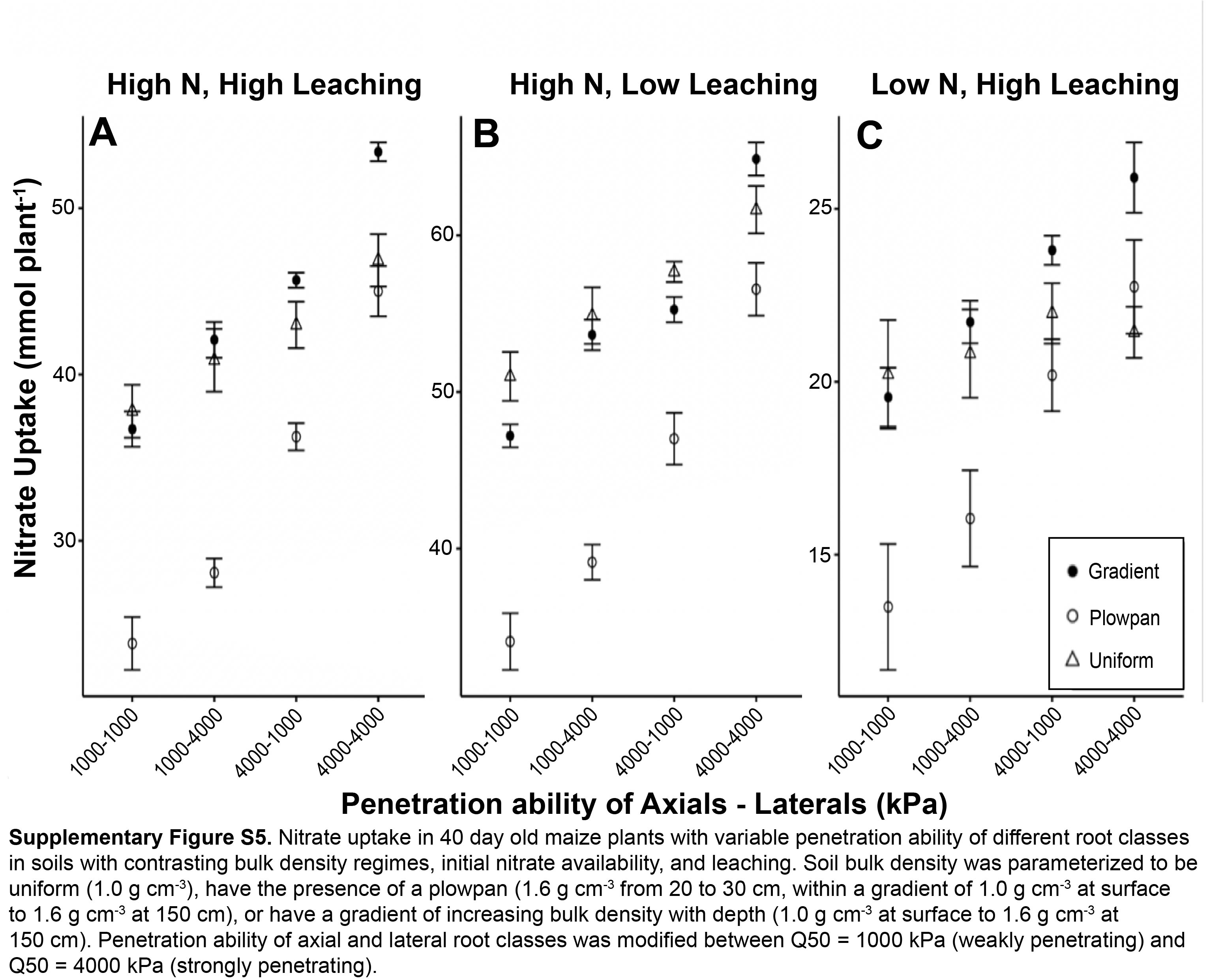

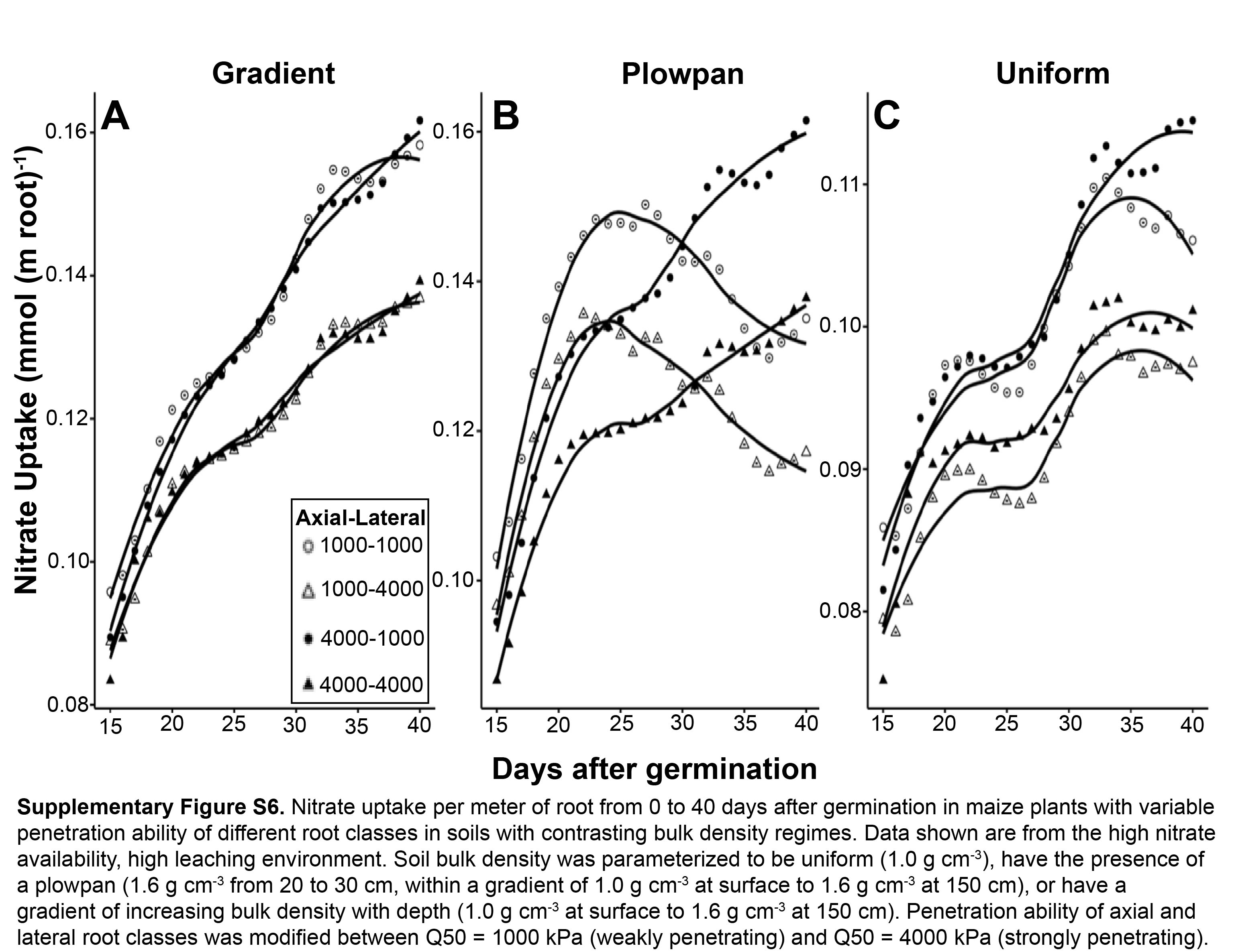

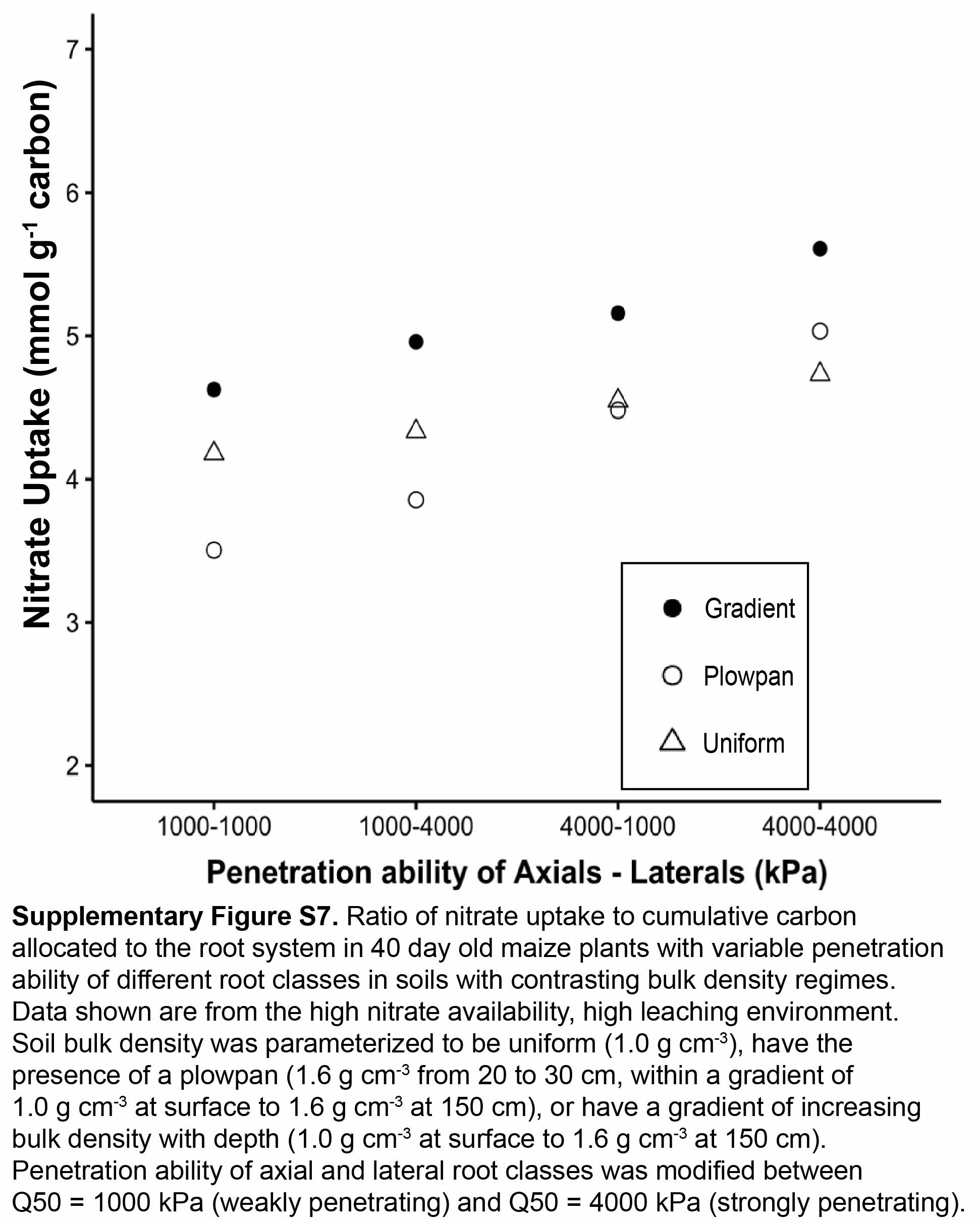

Supplement: mcab144_suppl_Supplementary_Figures [file mcab144_suppl_supplementary_figures.docx]
