# Supplementary material for: Theoretical evidence that root penetration ability interacts with soil compaction regimes to affect nitrate capture
Source: Ann Bot. 2021 Nov 30;129(3):315–30. doi: 10.1093/aob/mcab144 (PMC8835659; doi:10.1093/aob/mcab144)
Supplement: mcab144_suppl_Supplementary_Tables [file mcab144_suppl_supplementary_tables.docx]

**Supplementary Information**

**Supplementary Table S1.** Bulk density (d_b_) and the van Ganuchten parameters: residual water content (θ_r_), water content at saturation (θ_s_), and the saturated hydraulic conductivity (K_s_) used to parameterize the *gradient*, *plowpan*, and *uniform* soil profiles. α and n describe the soil-water retention curve.

| **Soil** | | **Depth** | **d_b_** | | | **θ_r_** | **θ_s_** | **α** | | **n** | **K_s_** |  |
| --- | --- | --- | --- | --- | --- | --- | --- | --- | --- | --- | --- | --- |
|  | | **(cm)** | **(g cm^-3^)** | | | **(cm^3^ cm^-3^)** | **(cm^3^ cm^-3^)** | **(hPa^-1^)** | |  | **(cm d^-1^)** |  |
| Gradient | 0 | | | 1 | 0.0875 | | 0.5482 | 0.0059 | 1.6140 | | 89.2100 | |
| Gradient | -1 | | | 1.004 | 0.0874 | | 0.5469 | 0.0059 | 1.6144 | | 87.7253 | |
| Gradient | -2 | | | 1.008 | 0.0872 | | 0.5456 | 0.0059 | 1.6148 | | 86.2609 | |
| Gradient | -3 | | | 1.012 | 0.0871 | | 0.5443 | 0.0058 | 1.6152 | | 84.8165 | |
| Gradient | -4 | | | 1.016 | 0.0870 | | 0.5431 | 0.0058 | 1.6156 | | 83.3920 | |
| Gradient | -5 | | | 1.02 | 0.0869 | | 0.5418 | 0.0058 | 1.6160 | | 81.9873 | |
| Gradient | -6 | | | 1.024 | 0.0867 | | 0.5405 | 0.0058 | 1.6164 | | 80.6022 | |
| Gradient | -7 | | | 1.028 | 0.0866 | | 0.5392 | 0.0058 | 1.6168 | | 79.2366 | |
| Gradient | -8 | | | 1.032 | 0.0865 | | 0.5380 | 0.0058 | 1.6171 | | 77.8902 | |
| Gradient | -9 | | | 1.036 | 0.0864 | | 0.5367 | 0.0058 | 1.6175 | | 76.5629 | |
| Gradient | -10 | | | 1.04 | 0.0862 | | 0.5355 | 0.0058 | 1.6179 | | 75.2547 | |
| Gradient | -11 | | | 1.044 | 0.0861 | | 0.5342 | 0.0058 | 1.6182 | | 73.9652 | |
| Gradient | -12 | | | 1.048 | 0.0860 | | 0.5330 | 0.0058 | 1.6186 | | 72.6943 | |
| Gradient | -13 | | | 1.052 | 0.0859 | | 0.5317 | 0.0058 | 1.6189 | | 71.4419 | |
| Gradient | -14 | | | 1.056 | 0.0858 | | 0.5305 | 0.0058 | 1.6193 | | 70.2077 | |
| Gradient | -15 | | | 1.06 | 0.0856 | | 0.5293 | 0.0058 | 1.6196 | | 68.9917 | |
| Gradient | -16 | | | 1.064 | 0.0855 | | 0.5281 | 0.0057 | 1.6199 | | 67.7936 | |
| Gradient | -17 | | | 1.068 | 0.0854 | | 0.5269 | 0.0057 | 1.6202 | | 66.6132 | |
| Gradient | -18 | | | 1.072 | 0.0853 | | 0.5256 | 0.0057 | 1.6206 | | 65.4505 | |
| Gradient | -19 | | | 1.076 | 0.0852 | | 0.5244 | 0.0057 | 1.6209 | | 64.3051 | |
| Gradient | -20 | | | 1.08 | 0.0851 | | 0.5232 | 0.0057 | 1.6212 | | 63.1770 | |
| Gradient | -21 | | | 1.084 | 0.0850 | | 0.5220 | 0.0057 | 1.6215 | | 62.0659 | |
| Gradient | -22 | | | 1.088 | 0.0849 | | 0.5209 | 0.0057 | 1.6217 | | 60.9717 | |
| Gradient | -23 | | | 1.092 | 0.0847 | | 0.5197 | 0.0057 | 1.6220 | | 59.8942 | |
| Gradient | -24 | | | 1.096 | 0.0846 | | 0.5185 | 0.0057 | 1.6223 | | 58.8332 | |
| Gradient | -25 | | | 1.1 | 0.0845 | | 0.5173 | 0.0057 | 1.6226 | | 57.7886 | |
| Gradient | -26 | | | 1.104 | 0.0844 | | 0.5161 | 0.0057 | 1.6228 | | 56.7601 | |
| Gradient | -27 | | | 1.108 | 0.0843 | | 0.5150 | 0.0057 | 1.6231 | | 55.7475 | |
| Gradient | -28 | | | 1.112 | 0.0842 | | 0.5138 | 0.0057 | 1.6233 | | 54.7508 | |
| Gradient | -29 | | | 1.116 | 0.0841 | | 0.5126 | 0.0057 | 1.6236 | | 53.7697 | |
| Gradient | -30 | | | 1.12 | 0.0840 | | 0.5115 | 0.0057 | 1.6238 | | 52.8041 | |
| Gradient | -31 | | | 1.124 | 0.0839 | | 0.5103 | 0.0057 | 1.6241 | | 51.8537 | |
| Gradient | -32 | | | 1.128 | 0.0838 | | 0.5092 | 0.0057 | 1.6243 | | 50.9185 | |
| Gradient | -33 | | | 1.132 | 0.0837 | | 0.5081 | 0.0057 | 1.6245 | | 49.9981 | |
| Gradient | -34 | | | 1.136 | 0.0836 | | 0.5069 | 0.0057 | 1.6247 | | 49.0925 | |
| Gradient | -35 | | | 1.14 | 0.0835 | | 0.5058 | 0.0057 | 1.6249 | | 48.2014 | |
| Gradient | -36 | | | 1.144 | 0.0834 | | 0.5047 | 0.0057 | 1.6251 | | 47.3248 | |
| Gradient | -37 | | | 1.148 | 0.0833 | | 0.5035 | 0.0057 | 1.6253 | | 46.4624 | |
| Gradient | -38 | | | 1.152 | 0.0832 | | 0.5024 | 0.0057 | 1.6255 | | 45.6140 | |
| Gradient | -39 | | | 1.156 | 0.0831 | | 0.5013 | 0.0057 | 1.6256 | | 44.7795 | |
| Gradient | -40 | | | 1.16 | 0.0830 | | 0.5002 | 0.0057 | 1.6258 | | 43.9587 | |
| Gradient | -41 | | | 1.164 | 0.0829 | | 0.4991 | 0.0057 | 1.6260 | | 43.1514 | |
| Gradient | -42 | | | 1.168 | 0.0828 | | 0.4980 | 0.0057 | 1.6261 | | 42.3575 | |
| Gradient | -43 | | | 1.172 | 0.0827 | | 0.4969 | 0.0057 | 1.6263 | | 41.5768 | |
| Gradient | -44 | | | 1.176 | 0.0826 | | 0.4958 | 0.0057 | 1.6264 | | 40.8091 | |
| Gradient | -45 | | | 1.18 | 0.0825 | | 0.4947 | 0.0057 | 1.6265 | | 40.0543 | |
| Gradient | -46 | | | 1.184 | 0.0824 | | 0.4936 | 0.0057 | 1.6266 | | 39.3122 | |
| Gradient | -47 | | | 1.188 | 0.0823 | | 0.4925 | 0.0057 | 1.6267 | | 38.5826 | |
| Gradient | -48 | | | 1.192 | 0.0822 | | 0.4914 | 0.0057 | 1.6269 | | 37.8654 | |
| Gradient | -49 | | | 1.196 | 0.0821 | | 0.4904 | 0.0057 | 1.6269 | | 37.1604 | |
| Gradient | -50 | | | 1.2 | 0.0820 | | 0.4893 | 0.0057 | 1.6270 | | 36.4674 | |
| Gradient | -51 | | | 1.204 | 0.0819 | | 0.4882 | 0.0057 | 1.6271 | | 35.7863 | |
| Gradient | -52 | | | 1.208 | 0.0818 | | 0.4871 | 0.0057 | 1.6272 | | 35.1170 | |
| Gradient | -53 | | | 1.212 | 0.0817 | | 0.4861 | 0.0057 | 1.6272 | | 34.4592 | |
| Gradient | -54 | | | 1.216 | 0.0816 | | 0.4850 | 0.0057 | 1.6273 | | 33.8128 | |
| Gradient | -55 | | | 1.22 | 0.0815 | | 0.4840 | 0.0057 | 1.6273 | | 33.1777 | |
| Gradient | -56 | | | 1.224 | 0.0814 | | 0.4829 | 0.0057 | 1.6274 | | 32.5536 | |
| Gradient | -57 | | | 1.228 | 0.0813 | | 0.4818 | 0.0057 | 1.6274 | | 31.9405 | |
| Gradient | -58 | | | 1.232 | 0.0812 | | 0.4808 | 0.0057 | 1.6274 | | 31.3382 | |
| Gradient | -59 | | | 1.236 | 0.0812 | | 0.4797 | 0.0057 | 1.6274 | | 30.7464 | |
| Gradient | -60 | | | 1.24 | 0.0811 | | 0.4787 | 0.0057 | 1.6274 | | 30.1652 | |
| Gradient | -61 | | | 1.244 | 0.0810 | | 0.4777 | 0.0057 | 1.6274 | | 29.5943 | |
| Gradient | -62 | | | 1.248 | 0.0809 | | 0.4766 | 0.0057 | 1.6274 | | 29.0336 | |
| Gradient | -63 | | | 1.252 | 0.0808 | | 0.4756 | 0.0057 | 1.6273 | | 28.4829 | |
| Gradient | -64 | | | 1.256 | 0.0807 | | 0.4746 | 0.0057 | 1.6273 | | 27.9420 | |
| Gradient | -65 | | | 1.26 | 0.0806 | | 0.4735 | 0.0057 | 1.6272 | | 27.4109 | |
| Gradient | -66 | | | 1.264 | 0.0805 | | 0.4725 | 0.0057 | 1.6271 | | 26.8894 | |
| Gradient | -67 | | | 1.268 | 0.0804 | | 0.4715 | 0.0057 | 1.6271 | | 26.3774 | |
| Gradient | -68 | | | 1.272 | 0.0803 | | 0.4705 | 0.0057 | 1.6270 | | 25.8746 | |
| Gradient | -69 | | | 1.276 | 0.0802 | | 0.4694 | 0.0057 | 1.6269 | | 25.3810 | |
| Gradient | -70 | | | 1.28 | 0.0801 | | 0.4684 | 0.0057 | 1.6268 | | 24.8963 | |
| Gradient | -71 | | | 1.284 | 0.0800 | | 0.4674 | 0.0057 | 1.6266 | | 24.4206 | |
| Gradient | -72 | | | 1.288 | 0.0800 | | 0.4664 | 0.0057 | 1.6265 | | 23.9536 | |
| Gradient | -73 | | | 1.292 | 0.0799 | | 0.4654 | 0.0057 | 1.6263 | | 23.4952 | |
| Gradient | -74 | | | 1.296 | 0.0798 | | 0.4644 | 0.0057 | 1.6262 | | 23.0452 | |
| Gradient | -75 | | | 1.3 | 0.0797 | | 0.4634 | 0.0057 | 1.6260 | | 22.6036 | |
| Gradient | -76 | | | 1.304 | 0.0796 | | 0.4624 | 0.0057 | 1.6258 | | 22.1702 | |
| Gradient | -77 | | | 1.308 | 0.0795 | | 0.4614 | 0.0057 | 1.6256 | | 21.7448 | |
| Gradient | -78 | | | 1.312 | 0.0794 | | 0.4604 | 0.0057 | 1.6254 | | 21.3273 | |
| Gradient | -79 | | | 1.316 | 0.0793 | | 0.4594 | 0.0057 | 1.6252 | | 20.9177 | |
| Gradient | -80 | | | 1.32 | 0.0792 | | 0.4584 | 0.0057 | 1.6249 | | 20.5157 | |
| Gradient | -81 | | | 1.324 | 0.0791 | | 0.4574 | 0.0057 | 1.6247 | | 20.1212 | |
| Gradient | -82 | | | 1.328 | 0.0790 | | 0.4564 | 0.0058 | 1.6244 | | 19.7342 | |
| Gradient | -83 | | | 1.332 | 0.0789 | | 0.4554 | 0.0058 | 1.6241 | | 19.3544 | |
| Gradient | -84 | | | 1.336 | 0.0788 | | 0.4544 | 0.0058 | 1.6238 | | 18.9819 | |
| Gradient | -85 | | | 1.34 | 0.0787 | | 0.4535 | 0.0058 | 1.6235 | | 18.6163 | |
| Gradient | -86 | | | 1.344 | 0.0786 | | 0.4525 | 0.0058 | 1.6232 | | 18.2577 | |
| Gradient | -87 | | | 1.348 | 0.0785 | | 0.4515 | 0.0058 | 1.6229 | | 17.9059 | |
| Gradient | -88 | | | 1.352 | 0.0784 | | 0.4505 | 0.0058 | 1.6225 | | 17.5608 | |
| Gradient | -89 | | | 1.356 | 0.0784 | | 0.4495 | 0.0058 | 1.6221 | | 17.2223 | |
| Gradient | -90 | | | 1.36 | 0.0783 | | 0.4486 | 0.0058 | 1.6217 | | 16.8902 | |
| Gradient | -91 | | | 1.364 | 0.0782 | | 0.4476 | 0.0058 | 1.6213 | | 16.5645 | |
| Gradient | -92 | | | 1.368 | 0.0781 | | 0.4466 | 0.0058 | 1.6209 | | 16.2450 | |
| Gradient | -93 | | | 1.372 | 0.0780 | | 0.4457 | 0.0058 | 1.6205 | | 15.9316 | |
| Gradient | -94 | | | 1.376 | 0.0779 | | 0.4447 | 0.0058 | 1.6200 | | 15.6242 | |
| Gradient | -95 | | | 1.38 | 0.0778 | | 0.4437 | 0.0058 | 1.6195 | | 15.3228 | |
| Gradient | -96 | | | 1.384 | 0.0777 | | 0.4428 | 0.0058 | 1.6190 | | 15.0272 | |
| Gradient | -97 | | | 1.388 | 0.0776 | | 0.4418 | 0.0059 | 1.6185 | | 14.7372 | |
| Gradient | -98 | | | 1.392 | 0.0775 | | 0.4408 | 0.0059 | 1.6180 | | 14.4529 | |
| Gradient | -99 | | | 1.396 | 0.0774 | | 0.4399 | 0.0059 | 1.6174 | | 14.1740 | |
| Gradient | -100 | | | 1.4 | 0.0773 | | 0.4389 | 0.0059 | 1.6169 | | 13.9005 | |
| Gradient | -101 | | | 1.404 | 0.0772 | | 0.4380 | 0.0059 | 1.6163 | | 13.6324 | |
| Gradient | -102 | | | 1.408 | 0.0771 | | 0.4370 | 0.0059 | 1.6157 | | 13.3694 | |
| Gradient | -103 | | | 1.412 | 0.0770 | | 0.4360 | 0.0059 | 1.6151 | | 13.1115 | |
| Gradient | -104 | | | 1.416 | 0.0768 | | 0.4351 | 0.0059 | 1.6144 | | 12.8587 | |
| Gradient | -105 | | | 1.42 | 0.0767 | | 0.4341 | 0.0059 | 1.6137 | | 12.6107 | |
| Gradient | -106 | | | 1.424 | 0.0766 | | 0.4332 | 0.0059 | 1.6131 | | 12.3676 | |
| Gradient | -107 | | | 1.428 | 0.0765 | | 0.4322 | 0.0060 | 1.6124 | | 12.1292 | |
| Gradient | -108 | | | 1.432 | 0.0764 | | 0.4313 | 0.0060 | 1.6116 | | 11.8955 | |
| Gradient | -109 | | | 1.436 | 0.0763 | | 0.4303 | 0.0060 | 1.6109 | | 11.6663 | |
| Gradient | -110 | | | 1.44 | 0.0762 | | 0.4294 | 0.0060 | 1.6101 | | 11.4416 | |
| Gradient | -111 | | | 1.444 | 0.0761 | | 0.4284 | 0.0060 | 1.6093 | | 11.2213 | |
| Gradient | -112 | | | 1.448 | 0.0760 | | 0.4275 | 0.0060 | 1.6085 | | 11.0053 | |
| Gradient | -113 | | | 1.452 | 0.0759 | | 0.4265 | 0.0060 | 1.6076 | | 10.7935 | |
| Gradient | -114 | | | 1.456 | 0.0758 | | 0.4256 | 0.0060 | 1.6068 | | 10.5859 | |
| Gradient | -115 | | | 1.46 | 0.0756 | | 0.4246 | 0.0060 | 1.6059 | | 10.3823 | |
| Gradient | -116 | | | 1.464 | 0.0755 | | 0.4237 | 0.0061 | 1.6049 | | 10.1828 | |
| Gradient | -117 | | | 1.468 | 0.0754 | | 0.4227 | 0.0061 | 1.6040 | | 9.9871 | |
| Gradient | -118 | | | 1.472 | 0.0753 | | 0.4218 | 0.0061 | 1.6030 | | 9.7953 | |
| Gradient | -119 | | | 1.476 | 0.0752 | | 0.4209 | 0.0061 | 1.6020 | | 9.6073 | |
| Gradient | -120 | | | 1.48 | 0.0751 | | 0.4199 | 0.0061 | 1.6010 | | 9.4229 | |
| Gradient | -121 | | | 1.484 | 0.0749 | | 0.4190 | 0.0061 | 1.6000 | | 9.2422 | |
| Gradient | -122 | | | 1.488 | 0.0748 | | 0.4180 | 0.0061 | 1.5989 | | 9.0650 | |
| Gradient | -123 | | | 1.492 | 0.0747 | | 0.4171 | 0.0062 | 1.5978 | | 8.8913 | |
| Gradient | -124 | | | 1.496 | 0.0746 | | 0.4161 | 0.0062 | 1.5967 | | 8.7211 | |
| Gradient | -125 | | | 1.5 | 0.0745 | | 0.4152 | 0.0062 | 1.5955 | | 8.5542 | |
| Gradient | -126 | | | 1.504 | 0.0743 | | 0.4142 | 0.0062 | 1.5943 | | 8.3905 | |
| Gradient | -127 | | | 1.508 | 0.0742 | | 0.4133 | 0.0062 | 1.5931 | | 8.2301 | |
| Gradient | -128 | | | 1.512 | 0.0741 | | 0.4124 | 0.0062 | 1.5918 | | 8.0729 | |
| Gradient | -129 | | | 1.516 | 0.0739 | | 0.4114 | 0.0062 | 1.5906 | | 7.9188 | |
| Gradient | -130 | | | 1.52 | 0.0738 | | 0.4105 | 0.0063 | 1.5892 | | 7.7677 | |
| Gradient | -131 | | | 1.524 | 0.0737 | | 0.4095 | 0.0063 | 1.5879 | | 7.6196 | |
| Gradient | -132 | | | 1.528 | 0.0735 | | 0.4086 | 0.0063 | 1.5865 | | 7.4744 | |
| Gradient | -133 | | | 1.532 | 0.0734 | | 0.4076 | 0.0063 | 1.5851 | | 7.3321 | |
| Gradient | -134 | | | 1.536 | 0.0733 | | 0.4067 | 0.0063 | 1.5837 | | 7.1927 | |
| Gradient | -135 | | | 1.54 | 0.0731 | | 0.4058 | 0.0064 | 1.5822 | | 7.0559 | |
| Gradient | -136 | | | 1.544 | 0.0730 | | 0.4048 | 0.0064 | 1.5807 | | 6.9219 | |
| Gradient | -137 | | | 1.548 | 0.0728 | | 0.4039 | 0.0064 | 1.5792 | | 6.7905 | |
| Gradient | -138 | | | 1.552 | 0.0727 | | 0.4029 | 0.0064 | 1.5776 | | 6.6618 | |
| Gradient | -139 | | | 1.556 | 0.0726 | | 0.4020 | 0.0064 | 1.5760 | | 6.5355 | |
| Gradient | -140 | | | 1.56 | 0.0724 | | 0.4010 | 0.0065 | 1.5744 | | 6.4118 | |
| Gradient | -141 | | | 1.564 | 0.0723 | | 0.4001 | 0.0065 | 1.5727 | | 6.2906 | |
| Gradient | -142 | | | 1.568 | 0.0721 | | 0.3991 | 0.0065 | 1.5710 | | 6.1717 | |
| Gradient | -143 | | | 1.572 | 0.0720 | | 0.3982 | 0.0065 | 1.5692 | | 6.0552 | |
| Gradient | -144 | | | 1.576 | 0.0718 | | 0.3972 | 0.0065 | 1.5675 | | 5.9410 | |
| Gradient | -145 | | | 1.58 | 0.0717 | | 0.3963 | 0.0066 | 1.5656 | | 5.8291 | |
| Gradient | -146 | | | 1.584 | 0.0715 | | 0.3953 | 0.0066 | 1.5638 | | 5.7194 | |
| Gradient | -147 | | | 1.588 | 0.0713 | | 0.3944 | 0.0066 | 1.5619 | | 5.6118 | |
| Gradient | -148 | | | 1.592 | 0.0712 | | 0.3934 | 0.0066 | 1.5600 | | 5.5064 | |
| Gradient | -149 | | | 1.596 | 0.0710 | | 0.3925 | 0.0067 | 1.5580 | | 5.4031 | |
| Gradient | -150 | | | 1.6 | 0.0708 | | 0.3915 | 0.0067 | 1.5560 | | 5.3019 | |
| Plowpan | 0 | | | 1 | 0.0875 | | 0.5482 | 0.0059 | 1.6140 | | 89.2100 | |
| Plowpan | -1 | | | 1.004 | 0.0874 | | 0.5469 | 0.0059 | 1.6144 | | 87.7253 | |
| Plowpan | -2 | | | 1.008 | 0.0872 | | 0.5456 | 0.0059 | 1.6148 | | 86.2609 | |
| Plowpan | -3 | | | 1.012 | 0.0871 | | 0.5443 | 0.0058 | 1.6152 | | 84.8165 | |
| Plowpan | -4 | | | 1.016 | 0.0870 | | 0.5431 | 0.0058 | 1.6156 | | 83.3920 | |
| Plowpan | -5 | | | 1.02 | 0.0869 | | 0.5418 | 0.0058 | 1.6160 | | 81.9873 | |
| Plowpan | -6 | | | 1.024 | 0.0867 | | 0.5405 | 0.0058 | 1.6164 | | 80.6022 | |
| Plowpan | -7 | | | 1.028 | 0.0866 | | 0.5392 | 0.0058 | 1.6168 | | 79.2366 | |
| Plowpan | -8 | | | 1.032 | 0.0865 | | 0.5380 | 0.0058 | 1.6171 | | 77.8902 | |
| Plowpan | -9 | | | 1.036 | 0.0864 | | 0.5367 | 0.0058 | 1.6175 | | 76.5629 | |
| Plowpan | -10 | | | 1.04 | 0.0862 | | 0.5355 | 0.0058 | 1.6179 | | 75.2547 | |
| Plowpan | -11 | | | 1.044 | 0.0861 | | 0.5342 | 0.0058 | 1.6182 | | 73.9652 | |
| Plowpan | -12 | | | 1.048 | 0.0860 | | 0.5330 | 0.0058 | 1.6186 | | 72.6943 | |
| Plowpan | -13 | | | 1.052 | 0.0859 | | 0.5317 | 0.0058 | 1.6189 | | 71.4419 | |
| Plowpan | -14 | | | 1.056 | 0.0858 | | 0.5305 | 0.0058 | 1.6193 | | 70.2077 | |
| Plowpan | -15 | | | 1.06 | 0.0856 | | 0.5293 | 0.0058 | 1.6196 | | 68.9917 | |
| Plowpan | -16 | | | 1.064 | 0.0855 | | 0.5281 | 0.0057 | 1.6199 | | 67.7936 | |
| Plowpan | -17 | | | 1.068 | 0.0854 | | 0.5269 | 0.0057 | 1.6202 | | 66.6132 | |
| Plowpan | -18 | | | 1.072 | 0.0853 | | 0.5256 | 0.0057 | 1.6206 | | 65.4505 | |
| Plowpan | -19 | | | 1.076 | 0.0852 | | 0.5244 | 0.0057 | 1.6209 | | 64.3051 | |
| Plowpan | -20 | | | 1.6 | 0.0708 | | 0.3915 | 0.0067 | 1.5560 | | 5.3019 | |
| Plowpan | -21 | | | 1.6 | 0.0708 | | 0.3915 | 0.0067 | 1.5560 | | 5.3019 | |
| Plowpan | -22 | | | 1.6 | 0.0708 | | 0.3915 | 0.0067 | 1.5560 | | 5.3019 | |
| Plowpan | -23 | | | 1.6 | 0.0708 | | 0.3915 | 0.0067 | 1.5560 | | 5.3019 | |
| Plowpan | -24 | | | 1.6 | 0.0708 | | 0.3915 | 0.0067 | 1.5560 | | 5.3019 | |
| Plowpan | -25 | | | 1.6 | 0.0708 | | 0.3915 | 0.0067 | 1.5560 | | 5.3019 | |
| Plowpan | -26 | | | 1.6 | 0.0708 | | 0.3915 | 0.0067 | 1.5560 | | 5.3019 | |
| Plowpan | -27 | | | 1.6 | 0.0708 | | 0.3915 | 0.0067 | 1.5560 | | 5.3019 | |
| Plowpan | -28 | | | 1.6 | 0.0708 | | 0.3915 | 0.0067 | 1.5560 | | 5.3019 | |
| Plowpan | -29 | | | 1.6 | 0.0708 | | 0.3915 | 0.0067 | 1.5560 | | 5.3019 | |
| Plowpan | -30 | | | 1.6 | 0.0708 | | 0.3915 | 0.0067 | 1.5560 | | 5.3019 | |
| Plowpan | -31 | | | 1.124 | 0.0839 | | 0.5103 | 0.0057 | 1.6241 | | 51.8537 | |
| Plowpan | -32 | | | 1.128 | 0.0838 | | 0.5092 | 0.0057 | 1.6243 | | 50.9185 | |
| Plowpan | -33 | | | 1.132 | 0.0837 | | 0.5081 | 0.0057 | 1.6245 | | 49.9981 | |
| Plowpan | -34 | | | 1.136 | 0.0836 | | 0.5069 | 0.0057 | 1.6247 | | 49.0925 | |
| Plowpan | -35 | | | 1.14 | 0.0835 | | 0.5058 | 0.0057 | 1.6249 | | 48.2014 | |
| Plowpan | -36 | | | 1.144 | 0.0834 | | 0.5047 | 0.0057 | 1.6251 | | 47.3248 | |
| Plowpan | -37 | | | 1.148 | 0.0833 | | 0.5035 | 0.0057 | 1.6253 | | 46.4624 | |
| Plowpan | -38 | | | 1.152 | 0.0832 | | 0.5024 | 0.0057 | 1.6255 | | 45.6140 | |
| Plowpan | -39 | | | 1.156 | 0.0831 | | 0.5013 | 0.0057 | 1.6256 | | 44.7795 | |
| Plowpan | -40 | | | 1.16 | 0.0830 | | 0.5002 | 0.0057 | 1.6258 | | 43.9587 | |
| Plowpan | -41 | | | 1.164 | 0.0829 | | 0.4991 | 0.0057 | 1.6260 | | 43.1514 | |
| Plowpan | -42 | | | 1.168 | 0.0828 | | 0.4980 | 0.0057 | 1.6261 | | 42.3575 | |
| Plowpan | -43 | | | 1.172 | 0.0827 | | 0.4969 | 0.0057 | 1.6263 | | 41.5768 | |
| Plowpan | -44 | | | 1.176 | 0.0826 | | 0.4958 | 0.0057 | 1.6264 | | 40.8091 | |
| Plowpan | -45 | | | 1.18 | 0.0825 | | 0.4947 | 0.0057 | 1.6265 | | 40.0543 | |
| Plowpan | -46 | | | 1.184 | 0.0824 | | 0.4936 | 0.0057 | 1.6266 | | 39.3122 | |
| Plowpan | -47 | | | 1.188 | 0.0823 | | 0.4925 | 0.0057 | 1.6267 | | 38.5826 | |
| Plowpan | -48 | | | 1.192 | 0.0822 | | 0.4914 | 0.0057 | 1.6269 | | 37.8654 | |
| Plowpan | -49 | | | 1.196 | 0.0821 | | 0.4904 | 0.0057 | 1.6269 | | 37.1604 | |
| Plowpan | -50 | | | 1.2 | 0.0820 | | 0.4893 | 0.0057 | 1.6270 | | 36.4674 | |
| Plowpan | -51 | | | 1.204 | 0.0819 | | 0.4882 | 0.0057 | 1.6271 | | 35.7863 | |
| Plowpan | -52 | | | 1.208 | 0.0818 | | 0.4871 | 0.0057 | 1.6272 | | 35.1170 | |
| Plowpan | -53 | | | 1.212 | 0.0817 | | 0.4861 | 0.0057 | 1.6272 | | 34.4592 | |
| Plowpan | -54 | | | 1.216 | 0.0816 | | 0.4850 | 0.0057 | 1.6273 | | 33.8128 | |
| Plowpan | -55 | | | 1.22 | 0.0815 | | 0.4840 | 0.0057 | 1.6273 | | 33.1777 | |
| Plowpan | -56 | | | 1.224 | 0.0814 | | 0.4829 | 0.0057 | 1.6274 | | 32.5536 | |
| Plowpan | -57 | | | 1.228 | 0.0813 | | 0.4818 | 0.0057 | 1.6274 | | 31.9405 | |
| Plowpan | -58 | | | 1.232 | 0.0812 | | 0.4808 | 0.0057 | 1.6274 | | 31.3382 | |
| Plowpan | -59 | | | 1.236 | 0.0812 | | 0.4797 | 0.0057 | 1.6274 | | 30.7464 | |
| Plowpan | -60 | | | 1.24 | 0.0811 | | 0.4787 | 0.0057 | 1.6274 | | 30.1652 | |
| Plowpan | -61 | | | 1.244 | 0.0810 | | 0.4777 | 0.0057 | 1.6274 | | 29.5943 | |
| Plowpan | -62 | | | 1.248 | 0.0809 | | 0.4766 | 0.0057 | 1.6274 | | 29.0336 | |
| Plowpan | -63 | | | 1.252 | 0.0808 | | 0.4756 | 0.0057 | 1.6273 | | 28.4829 | |
| Plowpan | -64 | | | 1.256 | 0.0807 | | 0.4746 | 0.0057 | 1.6273 | | 27.9420 | |
| Plowpan | -65 | | | 1.26 | 0.0806 | | 0.4735 | 0.0057 | 1.6272 | | 27.4109 | |
| Plowpan | -66 | | | 1.264 | 0.0805 | | 0.4725 | 0.0057 | 1.6271 | | 26.8894 | |
| Plowpan | -67 | | | 1.268 | 0.0804 | | 0.4715 | 0.0057 | 1.6271 | | 26.3774 | |
| Plowpan | -68 | | | 1.272 | 0.0803 | | 0.4705 | 0.0057 | 1.6270 | | 25.8746 | |
| Plowpan | -69 | | | 1.276 | 0.0802 | | 0.4694 | 0.0057 | 1.6269 | | 25.3810 | |
| Plowpan | -70 | | | 1.28 | 0.0801 | | 0.4684 | 0.0057 | 1.6268 | | 24.8963 | |
| Plowpan | -71 | | | 1.284 | 0.0800 | | 0.4674 | 0.0057 | 1.6266 | | 24.4206 | |
| Plowpan | -72 | | | 1.288 | 0.0800 | | 0.4664 | 0.0057 | 1.6265 | | 23.9536 | |
| Plowpan | -73 | | | 1.292 | 0.0799 | | 0.4654 | 0.0057 | 1.6263 | | 23.4952 | |
| Plowpan | -74 | | | 1.296 | 0.0798 | | 0.4644 | 0.0057 | 1.6262 | | 23.0452 | |
| Plowpan | -75 | | | 1.3 | 0.0797 | | 0.4634 | 0.0057 | 1.6260 | | 22.6036 | |
| Plowpan | -76 | | | 1.304 | 0.0796 | | 0.4624 | 0.0057 | 1.6258 | | 22.1702 | |
| Plowpan | -77 | | | 1.308 | 0.0795 | | 0.4614 | 0.0057 | 1.6256 | | 21.7448 | |
| Plowpan | -78 | | | 1.312 | 0.0794 | | 0.4604 | 0.0057 | 1.6254 | | 21.3273 | |
| Plowpan | -79 | | | 1.316 | 0.0793 | | 0.4594 | 0.0057 | 1.6252 | | 20.9177 | |
| Plowpan | -80 | | | 1.32 | 0.0792 | | 0.4584 | 0.0057 | 1.6249 | | 20.5157 | |
| Plowpan | -81 | | | 1.324 | 0.0791 | | 0.4574 | 0.0057 | 1.6247 | | 20.1212 | |
| Plowpan | -82 | | | 1.328 | 0.0790 | | 0.4564 | 0.0058 | 1.6244 | | 19.7342 | |
| Plowpan | -83 | | | 1.332 | 0.0789 | | 0.4554 | 0.0058 | 1.6241 | | 19.3544 | |
| Plowpan | -84 | | | 1.336 | 0.0788 | | 0.4544 | 0.0058 | 1.6238 | | 18.9819 | |
| Plowpan | -85 | | | 1.34 | 0.0787 | | 0.4535 | 0.0058 | 1.6235 | | 18.6163 | |
| Plowpan | -86 | | | 1.344 | 0.0786 | | 0.4525 | 0.0058 | 1.6232 | | 18.2577 | |
| Plowpan | -87 | | | 1.348 | 0.0785 | | 0.4515 | 0.0058 | 1.6229 | | 17.9059 | |
| Plowpan | -88 | | | 1.352 | 0.0784 | | 0.4505 | 0.0058 | 1.6225 | | 17.5608 | |
| Plowpan | -89 | | | 1.356 | 0.0784 | | 0.4495 | 0.0058 | 1.6221 | | 17.2223 | |
| Plowpan | -90 | | | 1.36 | 0.0783 | | 0.4486 | 0.0058 | 1.6217 | | 16.8902 | |
| Plowpan | -91 | | | 1.364 | 0.0782 | | 0.4476 | 0.0058 | 1.6213 | | 16.5645 | |
| Plowpan | -92 | | | 1.368 | 0.0781 | | 0.4466 | 0.0058 | 1.6209 | | 16.2450 | |
| Plowpan | -93 | | | 1.372 | 0.0780 | | 0.4457 | 0.0058 | 1.6205 | | 15.9316 | |
| Plowpan | -94 | | | 1.376 | 0.0779 | | 0.4447 | 0.0058 | 1.6200 | | 15.6242 | |
| Plowpan | -95 | | | 1.38 | 0.0778 | | 0.4437 | 0.0058 | 1.6195 | | 15.3228 | |
| Plowpan | -96 | | | 1.384 | 0.0777 | | 0.4428 | 0.0058 | 1.6190 | | 15.0272 | |
| Plowpan | -97 | | | 1.388 | 0.0776 | | 0.4418 | 0.0059 | 1.6185 | | 14.7372 | |
| Plowpan | -98 | | | 1.392 | 0.0775 | | 0.4408 | 0.0059 | 1.6180 | | 14.4529 | |
| Plowpan | -99 | | | 1.396 | 0.0774 | | 0.4399 | 0.0059 | 1.6174 | | 14.1740 | |
| Plowpan | -100 | | | 1.4 | 0.0773 | | 0.4389 | 0.0059 | 1.6169 | | 13.9005 | |
| Plowpan | -101 | | | 1.404 | 0.0772 | | 0.4380 | 0.0059 | 1.6163 | | 13.6324 | |
| Plowpan | -102 | | | 1.408 | 0.0771 | | 0.4370 | 0.0059 | 1.6157 | | 13.3694 | |
| Plowpan | -103 | | | 1.412 | 0.0770 | | 0.4360 | 0.0059 | 1.6151 | | 13.1115 | |
| Plowpan | -104 | | | 1.416 | 0.0768 | | 0.4351 | 0.0059 | 1.6144 | | 12.8587 | |
| Plowpan | -105 | | | 1.42 | 0.0767 | | 0.4341 | 0.0059 | 1.6137 | | 12.6107 | |
| Plowpan | -106 | | | 1.424 | 0.0766 | | 0.4332 | 0.0059 | 1.6131 | | 12.3676 | |
| Plowpan | -107 | | | 1.428 | 0.0765 | | 0.4322 | 0.0060 | 1.6124 | | 12.1292 | |
| Plowpan | -108 | | | 1.432 | 0.0764 | | 0.4313 | 0.0060 | 1.6116 | | 11.8955 | |
| Plowpan | -109 | | | 1.436 | 0.0763 | | 0.4303 | 0.0060 | 1.6109 | | 11.6663 | |
| Plowpan | -110 | | | 1.44 | 0.0762 | | 0.4294 | 0.0060 | 1.6101 | | 11.4416 | |
| Plowpan | -111 | | | 1.444 | 0.0761 | | 0.4284 | 0.0060 | 1.6093 | | 11.2213 | |
| Plowpan | -112 | | | 1.448 | 0.0760 | | 0.4275 | 0.0060 | 1.6085 | | 11.0053 | |
| Plowpan | -113 | | | 1.452 | 0.0759 | | 0.4265 | 0.0060 | 1.6076 | | 10.7935 | |
| Plowpan | -114 | | | 1.456 | 0.0758 | | 0.4256 | 0.0060 | 1.6068 | | 10.5859 | |
| Plowpan | -115 | | | 1.46 | 0.0756 | | 0.4246 | 0.0060 | 1.6059 | | 10.3823 | |
| Plowpan | -116 | | | 1.464 | 0.0755 | | 0.4237 | 0.0061 | 1.6049 | | 10.1828 | |
| Plowpan | -117 | | | 1.468 | 0.0754 | | 0.4227 | 0.0061 | 1.6040 | | 9.9871 | |
| Plowpan | -118 | | | 1.472 | 0.0753 | | 0.4218 | 0.0061 | 1.6030 | | 9.7953 | |
| Plowpan | -119 | | | 1.476 | 0.0752 | | 0.4209 | 0.0061 | 1.6020 | | 9.6073 | |
| Plowpan | -120 | | | 1.48 | 0.0751 | | 0.4199 | 0.0061 | 1.6010 | | 9.4229 | |
| Plowpan | -121 | | | 1.484 | 0.0749 | | 0.4190 | 0.0061 | 1.6000 | | 9.2422 | |
| Plowpan | -122 | | | 1.488 | 0.0748 | | 0.4180 | 0.0061 | 1.5989 | | 9.0650 | |
| Plowpan | -123 | | | 1.492 | 0.0747 | | 0.4171 | 0.0062 | 1.5978 | | 8.8913 | |
| Plowpan | -124 | | | 1.496 | 0.0746 | | 0.4161 | 0.0062 | 1.5967 | | 8.7211 | |
| Plowpan | -125 | | | 1.5 | 0.0745 | | 0.4152 | 0.0062 | 1.5955 | | 8.5542 | |
| Plowpan | -126 | | | 1.504 | 0.0743 | | 0.4142 | 0.0062 | 1.5943 | | 8.3905 | |
| Plowpan | -127 | | | 1.508 | 0.0742 | | 0.4133 | 0.0062 | 1.5931 | | 8.2301 | |
| Plowpan | -128 | | | 1.512 | 0.0741 | | 0.4124 | 0.0062 | 1.5918 | | 8.0729 | |
| Plowpan | -129 | | | 1.516 | 0.0739 | | 0.4114 | 0.0062 | 1.5906 | | 7.9188 | |
| Plowpan | -130 | | | 1.52 | 0.0738 | | 0.4105 | 0.0063 | 1.5892 | | 7.7677 | |
| Plowpan | -131 | | | 1.524 | 0.0737 | | 0.4095 | 0.0063 | 1.5879 | | 7.6196 | |
| Plowpan | -132 | | | 1.528 | 0.0735 | | 0.4086 | 0.0063 | 1.5865 | | 7.4744 | |
| Plowpan | -133 | | | 1.532 | 0.0734 | | 0.4076 | 0.0063 | 1.5851 | | 7.3321 | |
| Plowpan | -134 | | | 1.536 | 0.0733 | | 0.4067 | 0.0063 | 1.5837 | | 7.1927 | |
| Plowpan | -135 | | | 1.54 | 0.0731 | | 0.4058 | 0.0064 | 1.5822 | | 7.0559 | |
| Plowpan | -136 | | | 1.544 | 0.0730 | | 0.4048 | 0.0064 | 1.5807 | | 6.9219 | |
| Plowpan | -137 | | | 1.548 | 0.0728 | | 0.4039 | 0.0064 | 1.5792 | | 6.7905 | |
| Plowpan | -138 | | | 1.552 | 0.0727 | | 0.4029 | 0.0064 | 1.5776 | | 6.6618 | |
| Plowpan | -139 | | | 1.556 | 0.0726 | | 0.4020 | 0.0064 | 1.5760 | | 6.5355 | |
| Plowpan | -140 | | | 1.56 | 0.0724 | | 0.4010 | 0.0065 | 1.5744 | | 6.4118 | |
| Plowpan | -141 | | | 1.564 | 0.0723 | | 0.4001 | 0.0065 | 1.5727 | | 6.2906 | |
| Plowpan | -142 | | | 1.568 | 0.0721 | | 0.3991 | 0.0065 | 1.5710 | | 6.1717 | |
| Plowpan | -143 | | | 1.572 | 0.0720 | | 0.3982 | 0.0065 | 1.5692 | | 6.0552 | |
| Plowpan | -144 | | | 1.576 | 0.0718 | | 0.3972 | 0.0065 | 1.5675 | | 5.9410 | |
| Plowpan | -145 | | | 1.58 | 0.0717 | | 0.3963 | 0.0066 | 1.5656 | | 5.8291 | |
| Plowpan | -146 | | | 1.584 | 0.0715 | | 0.3953 | 0.0066 | 1.5638 | | 5.7194 | |
| Plowpan | -147 | | | 1.588 | 0.0713 | | 0.3944 | 0.0066 | 1.5619 | | 5.6118 | |
| Plowpan | -148 | | | 1.592 | 0.0712 | | 0.3934 | 0.0066 | 1.5600 | | 5.5064 | |
| Plowpan | -149 | | | 1.596 | 0.0710 | | 0.3925 | 0.0067 | 1.5580 | | 5.4031 | |
| Plowpan | -150 | | | 1.6 | 0.0708 | | 0.3915 | 0.0067 | 1.5560 | | 5.3019 | |
| Uniform | 0 > -150 | | | 1 | 0.0875 | | 0.5482 | 0.0059 | 1.6140 | | 89.2100 | |

**Supplementary Table S2.** *OpenSimRoot* parameterization for nitrogen and leaching regimes. Nitrate represents the quantity of nitrate initially available at germination and precipitation represents the total quantity of precipitation experienced through 40 days of growth.

| Simulated environment | Nitrate (kg ha^-1^) | Precipitation (mm) |
| --- | --- | --- |
| High N, High Leaching | 322.5 | 124 |
| Low N, High Leaching | 107.5 | 124 |
| High N, Low Leaching | 322.5 | 62 |
